# The effects of neutrophil-generated hypochlorous acid and other hypohalous acids on host and pathogens

**DOI:** 10.1007/s00018-020-03591-y

**Published:** 2020-07-13

**Authors:** Agnes Ulfig, Lars I. Leichert

**Affiliations:** Ruhr University Bochum, Institute for Biochemistry and Pathobiochemistry-Microbial Biochemistry, Universitätsstrasse 150, 44780 Bochum, Germany

**Keywords:** Neutrophil, MPO, HOCl, N-Chlorination, Thiol oxidation, Inflammation

## Abstract

Neutrophils are predominant immune cells that protect the human body against infections by deploying sophisticated antimicrobial strategies including phagocytosis of bacteria and neutrophil extracellular trap (NET) formation. Here, we provide an overview of the mechanisms by which neutrophils kill exogenous pathogens before we focus on one particular weapon in their arsenal: the generation of the oxidizing hypohalous acids HOCl, HOBr and HOSCN during the so-called oxidative burst by the enzyme myeloperoxidase. We look at the effects of these hypohalous acids on biological systems in general and proteins in particular and turn our attention to bacterial strategies to survive HOCl stress. HOCl is a strong inducer of protein aggregation, which bacteria can counteract by chaperone-like holdases that bind unfolding proteins without the need for energy in the form of ATP. These chaperones are activated by HOCl through thiol oxidation (Hsp33) or N-chlorination of basic amino acid side-chains (RidA and CnoX) and contribute to bacterial survival during HOCl stress. However, neutrophil-generated hypohalous acids also affect the host system. Recent studies have shown that plasma proteins act not only as sinks for HOCl, but get actively transformed into modulators of the cellular immune response through N-chlorination. N-chlorinated serum albumin can prevent aggregation of proteins, stimulate immune cells, and act as a pro-survival factor for immune cells in the presence of cytotoxic antigens. Finally, we take a look at the emerging role of HOCl as a potential signaling molecule, particularly its role in neutrophil extracellular trap formation.

## Introduction

The immune system protects the body against infection and diseases. Neutrophils are the dominant leukocyte in the blood and a key component of the innate immune response. In response to injury or infection, neutrophils are the first immune cells recruited to the affected tissue, where they deploy a variety of highly microbicidal weapons against a broad range of pathogens. Once arrived, neutrophils actively phagocytize microbes or form neutrophil extracellular traps (NETs) to bind and eliminate exogenous invaders (recently reviewed in [1, 2]).

Pathogen killing is initiated by the assembly of the superoxide (O_2_·^−^)-generating NADPH oxidase complex at the phagosomal membrane for the production of reactive oxygen/nitrogen species (ROS/RNS) and concomitant delivery of the heme enzyme myeloperoxidase (MPO) and other antimicrobial enzymes into the phagosome. This process is called “respiratory burst” or “oxidative burst” and comprises a central component of the neutrophil’s arsenal against pathogens (extensively reviewed in [3–5]).

MPO, once released into phagosomal compartments, catalyzes the production of the hypohalous acids hypochlorous acid (HOCl), hypobromous acid (HOBr) and hypothiocyanous acid (HOSCN) from hydrogen peroxide (H_2_O_2_) and the respective halide or pseudohalide ions [6–8]. HOCl and HOBr are kinetically two of the most reactive species generated in vivo, both exhibiting strong oxidizing and halogenating abilities [9]. As their reactivity with biomolecules is orders of magnitude higher than that of peroxynitrite (ONOO^–^) and H_2_O_2_, these acids appear indispensable for fulfilling the primary function of neutrophils in host immune defense: to protect the body against infectious diseases.

Due to their high reactivity toward a variety of biological molecules, HOCl and HOBr effectively contribute to the killing of phagocytized pathogens by causing oxidative damage to their proteins [10], DNA [11, 12], and lipids [13]. Although these oxidants can principally target all components of a pathogen, the major mechanism of killing, particularly by HOCl, is thought to be through the oxidative unfolding and aggregation of their proteins [10]. Oxidative stress-induced protein aggregation results in the loss of function of numerous proteins. Thus, if left unchecked, it can lead to cytotoxicity and ultimately cell death.

During their evolution bacteria, and other pathogens developed different strategies to avoid the detrimental effects of accumulating misfolded proteins and maintain a functional proteome during oxidative stress. It is well-established that ATP-dependent molecular chaperones (e.g. GroEL/GroES or DnaK/DnaJ/GrpE system) can actively assist in correct protein folding and protect misfolded proteins from aggregation, while proteases degrade stably misfolded and aggregated protein species via ATP-dependent mechanisms (recently reviewed in [14]). Under severe hypohalite-induced oxidative stress, however, these defenses seem to be incapacitated and alternative strategies are needed. Evidence emerging during the last few years shows that specific redox-regulated proteins play a pivotal role in protecting bacterial cells from neutrophil-derived oxidative stress. To date a number of proteins have been identified, which, upon exposure to oxidizing agents, particularly HOCl, turn into general, ATP-independent and highly active chaperone-like holdases capable of protecting essential proteins against stress-induced aggregation [10, 15, 16]. In these (known) cases, the HOCl-induced repurposing of proteins is triggered by oxidation of their thiol residues or N-halogenation of their basic amino acids. The temporary employment of additional chaperones in response to oxidative stress provides an efficient mechanism to specifically counteract and resist the strong oxidizing properties of MPO-derived hypohalous acids during infection or inflammation.

The effects of these MPO-derived oxidants, however, are not in any way selective for the pathogens but act rather nonspecific; therefore, their generation is not without risk to the host. Elevated levels of MPO and excessive generation of its products, particularly at sites of acute inflammation, can evidentially lead to undesired, collateral host tissue damage (recently reviewed in [17–21]). During inflammatory processes, hypohalous acid production by MPO is accompanied by additional stresses, such as increased temperature (fever) and lowered pH, which are themselves capable of inducing protein misfolding. It is, therefore, little surprise that the inflammatory nature of a variety of diseases, e.g. cardiovascular disease, neurodegenerative disorders, rheumatoid arthritis, chronic kidney disease, and some cancers [17–23], leads to a pathology associated with accumulation of misfolded proteins in the affected tissue.

Due to their high abundance in blood and interstitial fluid, human serum albumin (HSA) and other plasma proteins were found to effectively scavenge hypohalous acids in the vicinity of infection or inflammation [24–30]. Exposure to high concentrations of these oxidants, particularly HOCl, usually leads to various modifications of the plasma proteins. The resulting products, generally termed “advanced oxidation protein products” (AOPPs), have, therefore, been acknowledged as in vivo markers of chronic inflammation [23].

Plasma proteins, however, are no longer considered just passive sinks for hypohalous acids. The role of AOPPs during infectious and inflammatory processes has been an active area of research for many years and they appear to be both protective and detrimental in their effects [22, 31–37]. For example, we and others found that HOCl-modified plasma proteins exhibit chaperone-like function and prevent aggregation of other proteins and accelerate the host immune response by activating immune cells at sites of infection [36–39]. Although the latter effect may allow for faster pathogen clearance due to increased ROS/RNS generation, such a positive feedback loop could also ultimately lead to chronic inflammation.

In this review, we will illustrate recent advances in our understanding of the role of neutrophils during inflammation and infection and will provide an overview of the mechanisms by which neutrophils kill exogenous pathogens before we focus on the antimicrobial and inflammatory effects of hypohalous acids produced by neutrophils in infected or inflamed tissues. We discuss the role of hypohalous acid-induced modifications on the function of pathogen and host proteins and describe, how some of these protein modifications confer survival advantages to bacteria while others modulate the host immune response to infection or inflammation. Finally, we review recent developments in the understanding of the role of hypohalous acids, and particularly HOCl, as potential cellular signaling molecules and their role in a range of physiological processes.

## Neutrophil killing strategies against pathogens

### Neutrophil life cycle

So-called neutrophils, or polymorphonuclear leukocytes (PMNs), are the predominant type of leukocytes in the blood, comprising ~ 50–70% of the total white blood cells in most mammals. Around 10^11^ neutrophils are produced daily in the bone marrow from hematopoietic stem cells in a process called “granulopoiesis”. Neutrophil progenitors proliferate and continue to develop until recruited into the blood [40]. Neutrophil homeostasis in the bone marrow is maintained through a tight regulation of their production, release into blood and clearance from circulation (recently reviewed in [4, 41]). For decades, neutrophils have been regarded as short-lived cells with a circulating lifespan of 6–8 h before they return to the bone marrow for clearance [42]. More recent studies, however, found that under homeostatic conditions, neutrophils may circulate in human blood for 5.4 days, far longer than previously thought [43, 44].

Generally, neutrophil life span within tissues is thought to be two- to threefold longer than in circulation. Particularly at sites of inflammation, neutrophils have been reported to survive for up to 7 days due to the inhibition of cell apoptosis, an effect triggered by various inflammatory stimuli such as cytokines, pathogen-associated and damage-associated molecular pattern molecules (PAMPs and DAMPs) or environmental factors [45–48]. Indeed, an abnormally prolonged neutrophil life span can be observed in patients with chronic inflammatory diseases, thereby increasing disease severity through the excessive generation of antimicrobial products which may be injurious to host tissues [49, 50]. To prevent excessive tissue damage, neutrophils must, therefore, be quickly removed from inflammatory sites. Once they have completed their functions and reach the end of their life span, neutrophils undergo apoptosis and then are eliminated locally by resident macrophages and dendritic cells through the process of phagocytosis [42, 51]. Senescent neutrophils in circulation, however, were found to return to the bone marrow for final clearance upon upregulating expression of the cytokine receptor CXCR4, a central regulator of neutrophil trafficking under homeostatic conditions [52].

### Neutrophil recruitment to sites of infection and inflammation

Neutrophil activation and migration across endothelium in response to pathogen invasion or tissue injury have been comprehensively reviewed recently [53–55]. Briefly, circulating neutrophils patrol the blood continuously, until they encounter pathogen-derived chemoattractants and inflammatory signals released by immune cells and non-hematopoietic epithelial and endothelial cells in response to tissue injury or infection. The first signals that lead to neutrophil recruitment to injured tissues are thought to be DAMPs secreted by damaged and necrotic cells [56, 57]. DAMPs can act as chemoattractants or induce the production of several proinflammatory cytokines such as IL-1β and TNF-α by innate immune cells, primarily macrophages and dendritic cells in surrounding tissues. These cytokines then create a chemokine gradient, through which neutrophils migrate to the affected tissue. In the setting of microbial infection, PAMPs derived from the invading microbes are recognized by pattern recognition receptors (PRRs) present in the cytosol or at the cell surface of macrophages and other innate immune cells. Upon PAMP recognition, PRRs trigger a myriad of intracellular signaling cascades ultimately leading to the expression of a broad range of proinflammatory molecules [55, 58].

These inflammatory signals attract more circulating neutrophils and activate vascular endothelial cells near the site of infection to express cellular adhesion molecules, including selectins, on their surface. These molecules cause the neutrophils to slow down, tether to and roll along the luminal surface of the endothelium [59–61]. With further stimulation, neutrophils adhere firmly to the vessel wall and spread on the endothelial cells [62, 63]. During inflammation, the endothelial barrier is compromised due to the opening of intercellular gaps, leading to an increased vascular permeability which allows leukocytes and plasma proteins to enter tissues [64]. Once they pass through the gaps between endothelial cells in a process known as diapedesis, neutrophils migrate up the chemoattractant gradient to the site of injury or infection [54]. The neutrophil-derived chemokines also lead to the recruitment of other types of immune cells, such as monocytes, macrophages and dendritic cells [65]. These other cells also produce chemokines that promote neutrophil survival and recruitment, thus providing a positive feedback loop that sustains the inflammatory response [42, 66, 67].

Several human pathogens, however, have developed an impressive range of strategies to prevent neutrophil recruitment and activation. *Staphylococcus aureus* or *Streptococcus* species, for instance, secrete virulence factors, which can inhibit neutrophil recruitment by blocking neutrophil receptors responsible for binding chemokines, DAMPs or PAMPs [68–70], or by degrading chemotactic factors [70], such as IL-8, which is released by epithelial and endothelial cells to promote neutrophil recruitment [71]. For a detailed review of these evasion mechanisms, please refer to [72–74].

### Neutrophil arsenal against pathogens: phagocytosis and the “respiratory burst”

**Fig. 1:**
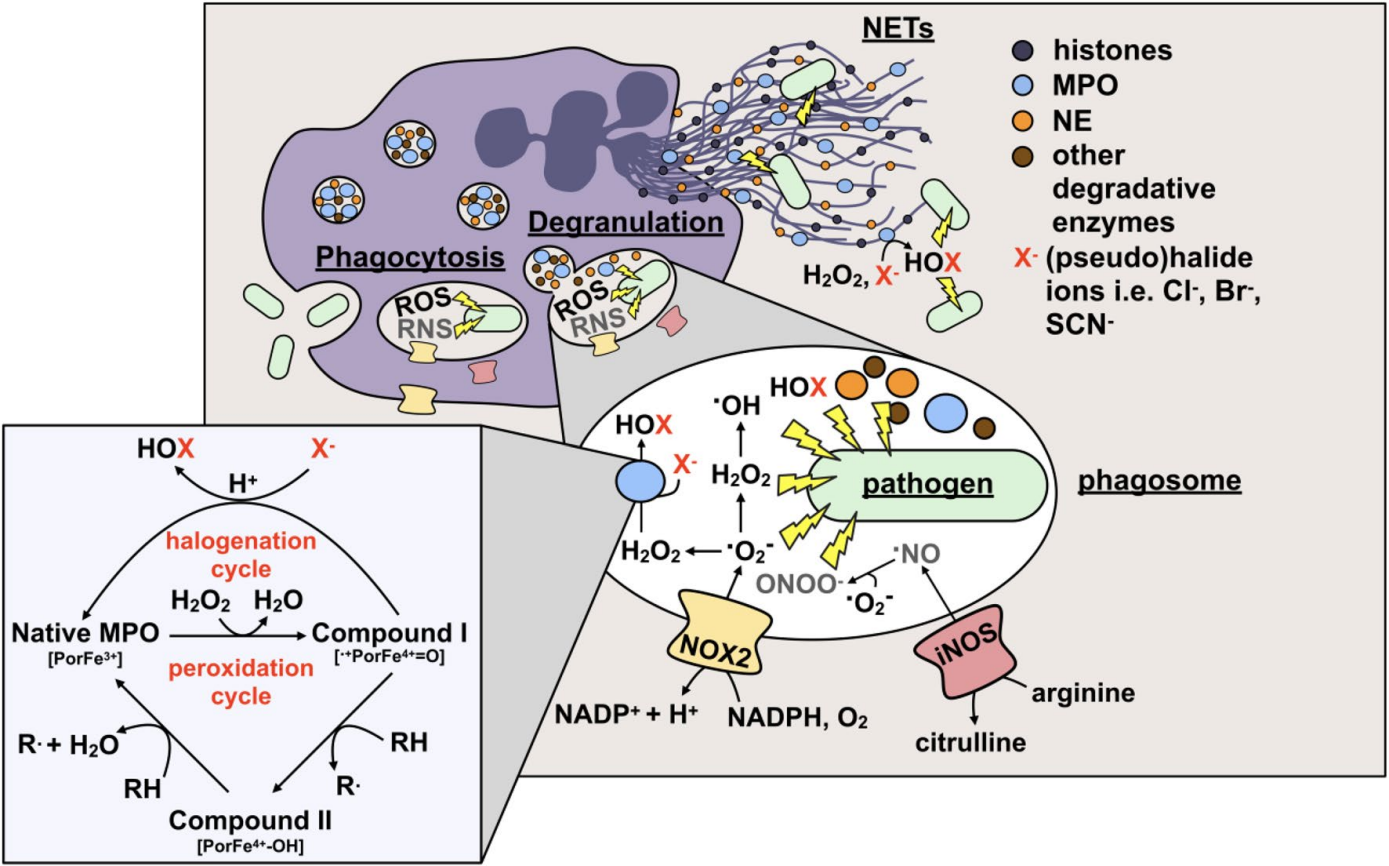
Neutrophil strategies to kill invading pathogens. Neutrophils are equipped with multiple weapons against pathogens, such as bacteria (light green) including uptake into phagosomes (phagocytosis). Subsequently, pathogens are degraded in the phagosome by several means. These include reactive oxygen and nitrogen species (ROS/RNS) generated by NADPH oxidase (NOX2) and inducible nitric oxide synthase (iNOS) as well as the release of antimicrobial effectors [i.e. neutrophil elastase (NE; orange), myeloperoxidase (MPO; blue), lysozyme and other degradative enzymes (brown)] into the phagosome (degranulation), and the formation of neutrophil extracellular traps (NETs). The NETs themselves are associated with antimicrobial proteins including histones, and the aforementioned NE and MPO. Inside the phagolysosome MPO reacts with H_2_O_2_ to form Compound I, the most oxidatively reactive state of MPO. Compound I can then react with an electron-rich organic substrate (RH) to form radical species (R·) and Compound II. Compound II subsequently reacts with another substrate (RH) to return to the native state of the MPO, completing the peroxidation cycle. But much more importantly, in the halogenation cycle (pseudo-)halide ions (X^−^) such as chloride (Cl^−^), bromide (Br^−^) or thiocyanate (SCN^−^) are oxidized by Compound I to yield the respective hypohalous acids (HOX), HOCl, HOBr or HOSCN, directly regenerating native MPO

When neutrophils are released into circulation, they are already fully equipped with an assortment of weapons against a wide range of infectious pathogens including bacteria, fungi and protozoan parasites [75]. If microorganisms manage to pass through the physical and chemical barriers provided by the skin, mucous membranes and endothelia throughout the human body, neutrophils become rapidly attracted to the site of infection. Once arrived, they kill pathogens through phagocytosis, production of ROS/RNS and the formation of NETs (Fig. 1).

Phagocytosis is a specific form of receptor-mediated endocytosis wherein neutrophils and other phagocytic immune cells engulf pathogens into a vacuole within the cell, the phagosome [76]. This process is most efficient in the presence of opsonins such as immunoglobulins (e.g. IgG) and complement factors, the predominant opsonins in serum.

Recognition of microbial pathogens is mediated by a diverse set of receptors present on the neutrophil surface, including PRRs (e.g. TLRs), G-protein-coupled receptors (GPCRs), and opsonic receptors (e.g. FcγR and complement receptors). These receptors recognize PAMPs, such as bacterial DNA or lipopolysaccharides (LPS), and host proteins that were used to opsonize the pathogen (e.g. IgG and complement).

When neutrophils ingest pathogenic invaders into phagosomes, they undergo a burst of oxygen consumption, also known as the “respiratory burst” (recently reviewed in [77]). This coincides with the release of a variety of antimicrobial effectors, including proteases, nucleases, antimicrobial peptides, lysozyme, and MPO, into the phagosomal lumen [78] (Fig. 1). The latter process, collectively termed phagosome “maturation”, involves a sequence of strictly coordinated membrane fusion and fission events between the phagosome and compartments of the endo/lysosomal network [79, 80] and ultimately culminates with the formation of the phagolysosome, a highly degradative organelle equipped with potent microbicidal properties [81, 82]. It has also been proposed that the neutrophil phagosome undergoes a progressive acidification during maturation similar to that of other phagocytes [83]. These measurements, however, utilized HOCl-reactive fluorescent dyes so that interference from MPO-catalyzed reactions cannot be excluded. More recent studies revealed that pH of neutrophil phagosomes remains rather unchanged over the duration of the respiratory burst or can even initially rise [84]. This defective acidification of neutrophil phagosomes is largely attributed to the reduced insertion of proton-pumping vacuolar-type (V-type) ATPases into the phagosomal membrane in the presence of an active NADPH oxidase [84] and the consumption of protons during dismutation of NADPH oxidase-derived superoxide, since the lack of NADPH oxidase seen in CGD patients or chemical inhibition of this enzyme led to a rapid and extensive fall in pH [85].

The increased utilization of oxygen by neutrophils during or following phagocytosis is mostly related to the assembly and activation of the NADPH oxidase (NOX2) in the plasma membrane, but more importantly in the phagosomal membrane (which is derived from the plasma membrane) [77]. Neutrophil NADPH oxidase, also commonly referred to as the phagocyte oxidase (Phox or NOX2) complex, is a multi-subunit enzyme comprising the cytosolic components p40*phox*, p47*phox*, p67*phox* and the membrane component flavocytochrome *b*_*558*_, a complex of gp91*phox* and p22*phox*. Upon stimulation, p47*phox* and p67*phox* form a complex that translocates to the plasma and/or phagosomal membrane, where it associates with flavocytochrome *b*_*558*_ to assemble the active oxidase. Activation also requires the participation of the small G-protein Rac 1/2 and Rap 1A. Aside from microorganisms, other stimuli such as phorbol-12-myristate-13-acetate (PMA) can also promote NOX2 assembly [86].

During phagocytosis, activation of the NADPH oxidase was found to occur mainly at the phagosomal membrane [87]. Active NOX2 moves electrons from cytosolic NADPH to oxygen to form highly unstable superoxide radical anions (O_2_·^−^) in the phagosomal lumen [88] (Fig. 1). Although O_2_·^−^ itself is poorly reactive with most biological substrates in aqueous environments [89], it serves as a progenitor for a number of other, more microbicidal ROS and plays a critical role in mediating a wide range of cellular signaling processes. Dismutation of O_2_·^−^ (either spontaneously or, at a significantly faster rate, through a reaction catalyzed by MPO itself [90]) gives rise to oxygen and H_2_O_2_, the latter being a precursor of one of the most powerful naturally occurring oxidants, the hydroxyl radical (·OH). However, in the phagosomal space, the H_2_O_2_ is mostly consumed by MPO for the oxidation of (pseudo-)halide ions (i.e. Cl^−^, Br^−^, SCN^−^) to the corresponding highly reactive hypohalous acids (HOCl, HOBr, HOSCN) (see below). Moreover, O_2_·^−^ can be protonated in the low pH of the phagocytic vacuole to form the more oxidizing hydroperoxyl radical HO_2_· (*E*°′ = 1.06 V for HO_2_· and *E*°′ = 0.94 V for O_2_·^−^) [91, 92]. In addition, O_2_·^−^ can react with equimolar concentrations of nitric oxide, synthesized by inducible nitric oxide synthase (iNOS), to produce the reactive nitrogen species peroxynitrite (OONO^−^). Once protonated, peroxynitrous acid (HOONO) can, albeit to a limited extent [93, 94], further decompose to ·OH and nitrogen dioxide (·NO_2_), both of which are more reactive than their common precursor [95–97]. The importance of O_2_·^−^ production for an effective antimicrobial and antifungal defense is best illustrated in chronic granulomatous disease (CGD), a primary immunodeficiency, where the lack of a functional NADPH oxidase results in recurrent infections and uncontrolled inflammatory responses due to the inability of neutrophils to generate oxidative metabolites [98–101].

Throughout the last two decades, there has been considerable debate about the role of ROS in eliminating pathogens by neutrophils [102, 103]. It is still controversial if ROS or microbicidal peptides and proteolytic enzymes are the more important components of the neutrophil antimicrobial arsenal [104, 105].

From the oxidative killing defect seen in CGD phagocytic cells, there is no doubt that ROS play an important role, but the actual mechanisms by which ROS damage pathogenic invaders in the phagosome are poorly understood [100]. Several lines of evidence suggest that ROS can contribute both directly and indirectly to killing by causing oxidative damage to various biomolecules or by stimulating pathogen elimination through various non-oxidative mechanisms [106].

The indirect role of ROS in promoting microbe clearance has been extensively reviewed [103] with a recent update of the literature [106, 107]. Briefly, ROS are produced not only in phagosomes during the phagocyte respiratory burst but also in other cell compartments, such as mitochondria or peroxisomes, as intermediaries in a number of different signal transduction pathways in the innate immune system [108], e.g. leukocyte PRR signaling. In addition, it has been demonstrated that ROS are also actively involved in the formation of NETs [109], autophagy [110–112], chemoattraction and activation of the inflammasome [113–115], programmed cell death of infected reservoirs [116, 117], antigen presentation, T-helper cell activation and lymphocyte proliferation [118–121]. Moreover, there is evidence suggesting that NADPH oxidase-dependent generation of ROS also plays a critical role in microbe killing by activating antimicrobial serine proteases and facilitating their release from the granules into the phagosome [105]. The lack of particular proteolytic enzymes has been reported to drastically impair both antibacterial and antifungal host defense, leading some investigators to postulate that activation of proteases is the major mechanism by which NADPH oxidase mediates host protection against infections [105, 122, 123]. It is still not fully understood how these various oxidative and non-oxidative mechanisms interconnect and there is conflicting data about which parts are just coincidental, which are necessary, and which are sufficient by themselves for effective pathogen elimination.

### Neutrophil extracellular traps (NETs)

NADPH oxidase-derived oxidants are thought to mediate activation of other neutrophil killing strategies against pathogens. It is commonly accepted that ROS are essential to initiate the formation of extracellular traps by activated neutrophils during infection and inflammation [124]. NETs represent a powerful and specific tool that allows neutrophils to capture and effectively destroy a broad range of pathogens while minimizing damage to host tissue [125]. They are characterized as extracellular fibrous structures composed of decondensed intracellular DNA associated with antimicrobial proteins such as neutrophil elastase (NE), lactoferrin, MPO, calprotectin and cathepsin G, and histones and some other cytoplasmic proteins [109, 126] (Fig. 1). All of these proteins can potentially kill or at least inhibit microorganisms by degrading virulence factors or disrupting their membrane integrity [127, 128]. Once released, NETs maintain a high concentration of these antimicrobial factors directly at the site of infection and support pathogen clearance. This is of particular importance, as in some cases neutrophils will no longer be able to produce ROS upon NET release. One enzyme that is thought to substantially contribute to NET antimicrobial activity is MPO. In vitro studies using isolated NETs revealed that MPO is present on NETs and exhibits significant activity upon addition of H_2_O_2_ [129, 130]. Assuming that there is enough extracellular H_2_O_2_ present at sites of infection, NET-bound MPO could generate reactive hypohalous acids in the immediate vicinity to trapped pathogens and thus effect their killing. However, direct experimental evidence that this, in fact, occurs in vivo is still lacking. The role of MPO and MPO-derived oxidants, particularly HOCl, in NET-mediated microbial killing will be discussed in detail later. Besides these antimicrobial properties, NETs were found to bind and trap microorganisms to reduce proliferation and prevent further spread of the pathogen in the body [109].

Since their discovery more than 15 years ago by Brinkmann and colleagues [109, 131], NETs have been the subject of extensive research in the field of innate immunity, but the molecular mechanisms behind NET formation are still not understood in detail.

First, NET formation was considered a particular form of cell death (“NETosis”) and thus, to be suicidal to neutrophils. This view, however, has been challenged by recent reports, which found that some neutrophils can survive this event and remain structurally intact, suggesting two mechanisms of NETosis: suicidal and vital [125, 131].

Suicidal and vital NETosis differ in their activation pathway and the nature of the stimulation. The mechanism of suicidal NETosis was found to be dependent on the activity of NADPH oxidase, NE, and MPO. It can be triggered by PMA [124, 132], IL-8, LPS [133] or different pathogens such as *Candida albicans* [134, 135]. In contrast, vital NETosis usually occurs independently of NADPH oxidase activity and is induced by some bacteria including *Escherichia coli* and *Staphylococcus aureus*, and bacteria-specific molecular patterns recognized by host PRRs, such as TLRs [132, 136]. Although both pathways have not been fully characterized yet, they appear to share similarities regarding the sequence of events leading to NET formation. In general, all forms of NETosis require intracellular membrane reorganization that allows the association of antimicrobial proteins from intracytoplasmic granules and chromatin to create NETs. Azurophilic granule proteins such as NE and MPO have to translocate to the nucleus to decondensate chromatin which then diffuses into the cytoplasm where additional antimicrobial and cytoplasmic proteins are attached to form early-stage NETs. The final result of the NET formation process depends on whether the suicidal or vital NETosis pathway has been activated. In suicidal NETosis, intracellular NET formation is followed by the rupture of the cell envelope resulting in the NET release into the extracellular surroundings, but also in neutrophil death and the loss of viable cell functions, such as chemotaxis and the ability to phagocytize pathogens. In contrast, vital NETosis ends up with the production of a DNA-filled vesicle that fuses with the outer membrane to release NETs [125, 131]. Since the plasma membrane remains intact, neutrophils that undergo vital NETosis remain temporarily functional as anuclear cytoplasts, still able to multitask. It is worth to emphasize that vital NETosis occurs completely independent of NADPH oxidase-mediated ROS generation and far more rapidly (5–15 min) compared with suicidal NETosis (1–4 h), which suggests different functions [137].

The strict dependence of suicidal NETosis on ROS generation by the NADPH oxidase has been demonstrated by some recent studies, which found that the absence of a functional NADPH oxidase in CGD patients or NOX2-deficient mice effectively suppressed NET formation [124, 135, 138]. Consistently, exogenous supplementation of H_2_O_2_ or reconstitution of NADPH oxidase function by gene therapy restored the ability of CGD neutrophils to produce NETs [124, 139]. In addition, some investigators observed a correlation between the level of NET formation, NET cell death and the amount of ROS produced, when they used different inbred mouse strains [135]. Although it is now commonly accepted that NADPH oxidase function is essential for suicidal NETosis, it is still unknown which ROS are involved downstream of the oxidase. Assigning the specific ROS required for NETosis is challenging as the site of NOX2 activation and the degree of degranulation, both of which affect the amount of the different ROS produced, vary depending on the stimulus. Hence, definite proof for many oxidants is still lacking.

Although generally considered important, directly NOX2-derived ROS are not the only crucial factor involved in the process of NETosis. For many years, NE and MPO have been also widely considered essential for death-mediated NETosis, but some aspects of their mechanisms of action are still unclear [140, 141]. This assumption, however, has been challenged by more recent studies demonstrating that NETosis can principally also occur in the absence of these enzymes and that their involvement depends on the nature of the stimuli that initiated the process of NET formation [134].

In response to ROS, NE leaves the azurophilic granule and translocates to the nucleus, where it initiates relaxation and decondensation of chromatin by cleaving histones, a crucial event in NET formation. Importance of NE has been demonstrated in a study by Papayannopoulos et al., where pharmacological inhibition of NE activity led to a complete block of NETosis and mice lacking NE also did not form NETs in a pulmonary model of *Klebsiella pneumoniae* infection [140]. On the other hand, it was recently reported that NE-deficient mice are still able to efficiently form NETs in response to non-infectious stimuli in vitro, indicating that NE may be not essential for NETosis per se [142]. Apparently, there are still a lot of discrepancies concerning the role of azurophilic enzymes in NET formation.

Several bacterial pathogens, however, have evolved impressive mechanisms to suppress, escape and/or resist NETs (for a detailed review see [143]). These evasion strategies can be classified into three categories. First, NETosis can be inhibited by the pathogens through the downregulation of host immune responses (e.g. attenuation of ROS generation [144], degradation of inflammatory chemokines [71] or via induction of the NET-suppressive cytokine interleukin-10 [145]). Second, pathogens can release nucleases to degrade the DNA backbone of NETs, ultimately leading to NET destruction [146, 147]. Finally, pathogens can also resist the microbicidal components of NETs [148]. Antimicrobial peptides attached to NETs are mostly cationic, creating an electrostatic force that attracts bacteria due to their negatively-charged surface. Therefore, several bacterial species have either a polysaccharide capsule to mask the negatively charged surface or have developed the ability to modify their surface charge via specific enzymes [148].

### Role of myeloperoxidase: not only a cytotoxic weapon against invaders

The green heme protein MPO is one of the most abundantly expressed pro-inflammatory enzymes in neutrophils accounting for ~ 5% of their dry mass (~ 10 × 10^−6^ μg MPO/cell) [149, 150]. MPO is stored in large amounts in the matrix of azurophil (primary) granules, which subsequently fuse with the phagocytic compartment after pathogen internalization. With the common membrane ruptured, MPO and other contents of the granules are discharged into the forming phagolysosome, where they manifest their antimicrobial potential toward a range of bacteria and fungi. While the majority of MPO remains in the phagolysosome, up to 30% of total cellular MPO can be secreted into the extracellular surroundings via degranulation, leakage during phagocytosis, or by association with NETs [129]. At sites of inflammation, the amount of MPO generated by accumulated phagocytes has been reported to reach a concentration of 1–2 mM [151–153]. The effects of elevated extracellular MPO levels on host cells and tissues are discussed later.

MPO, as a classical heme peroxidase, utilizes H_2_O_2_ to oxidize a variety of aromatic compounds (RH) by a 1-electron mechanism to give substrate radicals (R·) [154–156] (Fig. 1). The ability to generate the strong non-radical oxidant HOCl from H_2_O_2_ in the presence of chloride ions, however, has been thought to be unique to MPO among the mammalian heme peroxidases, serving as a biochemical fingerprint for the presence of enzymatically active MPO in tissue [157, 158]. Later observations expanded this view and showed that peroxidasin or its mammalian ortholog vascular peroxidase 1 (VPO1) [159] are other members of the heme peroxidase family, which are also capable of generating HOBr and HOCl, however, with significantly lower efficiency than MPO, providing a potential role for these peroxidases in innate immunity and host defense [160]. Even more important might be the recent finding, that peroxidasin also uses HOBr to form sulfilimine crosslinks in collagen IV scaffolds, a critical event for the assembly of basement membranes and tissue development [161]. This and the fact that chloride does not act as a two-electron donor of compound I in vitro in a truncated variant of human peroxidasin 1 suggests that HOBr is probably the relevant product of this protein in vivo [162–165].

MPO is also found in monocytes, however only at about one-third of the amount present in neutrophils [162–166]. Differentiation of monocytes to mature tissue macrophages is generally associated with a reduction of their microbicidal activity, partly due to a substantial decrease in oxygen-dependent mechanisms of toxicity leading to a much lower level of respiratory burst and MPO function [167–170]. However, significant amounts of MPO could be detected in various macrophage subpopulations (e.g. Kupffer cells of human liver [171], alveolar macrophages and microglia [172]), and in macrophages in human atherosclerotic lesions [163, 165]. Along this line, granulocyte–macrophage colony-stimulating factor (GM-CSF) has been found to regulate the ability of macrophages to express MPO and generate HOCl in vitro [173]. Alternatively, significant MPO activity in macrophages could also result from endocytosis of apoptotic neutrophils or the uptake of extracellular MPO [174]. These findings suggest that MPO is expressed and present in both neutrophils and macrophages throughout inflammation, albeit the MPO levels appear to vary dependent on the stage of inflammation: neutrophils were found to peak earlier, at the initial stage of inflammation, whereas in macrophages MPO was most abundant later [175].

Furthermore, it has been thought that only myeloid-lineage cells produce MPO, however, growing evidence suggests that MPO may also be a regular constituent of T lymphocytes [176] and B lymphocytes [177]. Thus, in addition to its known antimicrobial activity, MPO could have other, unanticipated cellular functions.

Since the discovery of MPO in the early 1970s as one of the granule enzymes being discharged into phagosomes by human neutrophils [178], there has been a surge of interest in elucidating the contribution of MPO-derived oxidants to the bactericidal and toxic properties of these cells. A plethora of studies showed that HOCl is the major strong oxidant produced by neutrophils and that it exhibits high activity against a wide range of bacterial, viral and fungal human pathogens, leading to the prevailing view that MPO is primarily responsible for phagocyte toxicity [179]. However, this concept has been challenged: more than 95% of individuals with hereditary MPO deficiency are asymptomatic and not at increased risk for life-threatening infectious complications suggesting that the MPO oxidant system is ancillary rather than essential for phagocyte-mediated microbicidal activity. Although MPO deficient neutrophils usually retain much of their ability to kill, they have been reported to have a pathogen killing time that is three to four times as long compared to neutrophils with functional MPO [180]. Moreover, in cases of fungal infection it has been shown that microbe clearance by MPO-deficient cells is much less efficient than that of normal neutrophils. One reason may be impaired or attenuated NET formation by neutrophils in the absence of MPO [130, 134, 140, 181]. In vitro, phagocytes deficient in MPO exhibit a severe defect in killing *C.* *albicans* and hyphal forms of *Aspergillus fumigatus*, and patients with hereditary MPO deficiency have an increased susceptibility to infections with these fungi [101, 182–185]. Together, one might conclude that action of MPO in innate host defense might be essential only in case of serious fungal infections and/or in situations where exposure of pathogens overwhelms the capacity of other host defense mechanisms. Along this line, recurrent severe infections with *C. albicans* have mostly been observed in patients who also suffered from other conditions, such as diabetes mellitus or cancer [186, 187].

The fact, that MPO deficient neutrophils are generally effective at killing microbes, albeit with a slower rate, indicates that the major NADPH oxidase products, superoxide and H_2_O_2_, must compensate for the lack of MPO and MPO-derived oxidants, and thus, be responsible for the observed killing activity. As they are not consumed by MPO, they will likely reach higher levels in the phagosomes of MPO-deficient neutrophils than in those of normal neutrophils [90]. Moreover, MPO deficient neutrophils have been reported to have a prolonged respiratory burst and an extended NADPH oxidase activity, leading to an increased production of superoxide and H_2_O_2_ [188–190]. Both oxidants are significantly less microbicidal compared to MPO-derived HOCl [191], providing a possible explanation for the delayed microbial killing by MPO deficient neutrophils.

Irrespective of the exact contribution of MPO to phagocyte toxicity, it is clear that highly efficient generation of reactive halogen species by MPO at sites of inflammation can drastically affect the function of both pathogen and host cells.

In the following, we will summarize current knowledge about the oxidative properties, target specificities and generated amounts of the MPO-derived hypohalous acids HOCl, HOBr and HOSCN, with a particular focus on their reaction with proteins, as those are the major targets of hypohalous acids under inflammatory conditions.

### Generation of hypohalous acids by myeloperoxidase

Activated neutrophils secrete MPO both into the phagosome and the extracellular environment (with the majority attached to NETs [129]).

Native MPO is a homodimer, consisting of two identical glycosylated protomers, each containing a light and a heavy chain, and a covalently bound modified heme [192–194]. The heavy chains of the two protomers are connected by a single disulfide bond [195]. The heme is a derivative of protoporphyrin IX, in which the methyl groups on pyrrole rings A and C are modified to allow the formation of ester linkages with the protein [192, 196]. The heme prosthetic group is covalently linked to the protein via autocatalytic formation of two ester bonds between modified methyl groups on pyrrole rings A and C and conserved aspartate (on the light chain) and glutamate residues (on the heavy chain) in MPO, and a sulfonium ion linkage between the vinyl group of pyrrole ring A and a heavy chain methionine [197–199]. These covalent linkages were found to be important in maintaining the catalytic activity of MPO, as replacement of MPO glutamate and methionine residues, that are involved in binding heme, strongly reduced the ability of MPO to catalyze the peroxidation of halide ions to hypohalous acids [200–202].

MPO catalyzes the reaction of halide and pseudohalide ions with hydrogen peroxide (H_2_O_2_) to form oxidizing hypohalous acids via the halogenation cycle [87, 170, 203] (Fig. 1). First, the native Fe(III) form of MPO reacts rapidly with H_2_O_2_ (with a rate of ~ 1.4 × 10^7^ M^−1^ s^−1^ [204]) to give the two-electron oxidized intermediate Compound I, a reactive Fe(IV) oxo porphyrin radical-cation species. Compound I can then undergo two-electron reduction with halide and pseudohalide ions (Cl^−^, Br^−^, SCN^−^) to generate the corresponding hypohalous acids HOCl, HOBr and HOSCN, thereby regenerating the Fe(III) (resting) state of MPO [7, 8, 205]. Among these halide and pseudohalide ions, SCN^−^ is the preferred substrate for MPO, as it has a much greater specificity constant (730:60:1 for SCN^−^, Br^−^ and Cl^−^ [8]) and reacts faster than either Cl^−^ or Br^−^ (rate constants *k* of 9.6 × 10^6^ M^−1^ s^−1^ for SCN^−^, 1.1 × 10^6^ M^−1^ s^−1^ for Br^−^ and 2.5 × 10^4^ M^−1^ s^−1^ for Cl^−^) [7]. However, Cl^−^ is a far more abundant MPO substrate, typically present in the plasma of healthy humans at a concentration of 100–140 mM [206, 207]. This is about two to three orders of magnitude higher than the concentration of Br^−^ and SCN^−^ (Br^−^, 20–100 μM; SCN^−^, 20–120 μM) [208, 209]. As a consequence, HOCl is typically the major reactive species formed by MPO under physiological conditions, while HOSCN and HOBr are produced in considerably lower amounts [8, 210, 211]. But there are numerous situations under which changes in plasma halide/pseudohalide concentrations occur, resulting in an altered extent of HOCl formation by MPO. While the concentration of Cl^−^ ions remains virtually unchanged in vivo due to the important role of this anion in maintaining ion gradients, the endogenous levels of Br^−^ and SCN^−^ have been reported to vary over a range of ~ fivefold and > tenfold, respectively [212]. Pathologically elevated concentrations of SCN^−^ can be typically found in individuals with a high intake of cyanide from tobacco smoking [212]. Elevated SCN^−^ has a much more marked effect on the HOCl: HOBr: HOSCN ratio than Br^−^, since thiocyanate is a better electron donor for MPO Compound I. Increasing levels of plasma SCN^−^ were found to decrease HOCl generation resulting in a changeover from HOCl as the major oxidizing agent (> 90% HOCl) to a mixture of HOCl and HOSCN. Up to 50% of the H_2_O_2_ consumed by MPO has been predicted to be converted to HOSCN under these conditions, with most of the remaining H_2_O_2_ (~ 45%) used to oxidize Cl^−^ anions to HOCl [210]. Furthermore, additional HOSCN can be generated in vivo by the direct reaction of SCN^−^ with HOCl and HOBr resulting in further decreased HOCl/HOBr plasma levels. Since this reaction is fast, particularly for HOBr (with a second-order rate constant *k* ≈ 2 × 10^7^ M^−1^ s^−1^ for HOCl [213] and *k* ≈ 2 × 10^9^ M^−1^ s^−1^ for HOBr [214]), SCN^−^ has been suggested to be the most effective endogenous scavenger of HOBr under biological conditions [214]. An altered ratio of hypohalous acid formation by MPO can markedly affect both the innate immune defense and the extent and nature of damage to host tissues. This is most likely due to the significant differences in reactivity and targets of the various hypohalous acids [212].

### Role of hypohalous acids in oxidative pathogen killing: proteins as major targets

MPO-mediated generation of halogenating oxidants within phagosomes is widely assumed to play a key role in bacterial cell killing and thus defending the body against disease [90, 215, 216]. HOCl is most commonly implicated as the reactive species responsible for neutrophil-mediated intracellular microbial killing (reviewed in [87, 150, 151]). HOCl is a strong oxidant (E^0^ [HOCl/Cl^−^] = + 1.28 V) and also the active ingredient of household bleach [217]. Other oxidants, such as O_2_·^−^ and H_2_O_2_, which are also generated within the phagosomal space, are orders of magnitude less microbicidal than HOCl and they are only effective in bacterial killing at much higher concentrations and/or upon long-term exposure. Thus, they appear to be of minor importance for the destruction of internalized pathogens [170, 218, 219]. While H_2_O_2_ has a substantially longer lifetime than HOCl under physiological conditions (10 μs [220] vs. 0.1 μs [221]) and can diffuse over considerable distances, readily passing membranes [222], HOCl appears to act locally and damage biomolecules within a radius of less than 0.1 μm [90, 221].

Conversion of long-lived and highly diffusible H_2_O_2_ into short-lived and locally confined HOCl by MPO thus provides a clever mechanism to specifically target pathogens within the phagosome and effectively protect neutrophil cytoplasm and surrounding host tissue against HOCl-induced oxidative damage. Restriction of HOCl to the phagosome within the neutrophil cell has been recently demonstrated by us using the genetically encoded redox sensor roGFP2 to monitor the redox state of neutrophil cytoplasm upon respiratory burst. Oxidation of the neutrophil cytosol was found to depend on active NADPH oxidase, but occurred independently of MPO activity, suggesting that in contrast to H_2_O_2_, HOCl is in fact unable to significantly permeate the phagosomal membrane during phagocytosis and thus remains in the immediate vicinity of the engulfed pathogen [132]. Using the same roGFP2-based probes in bacteria, we found that HOCl is indeed the major oxidant responsible for the oxidation of the cytoplasm of phagocytized bacteria [179].

The antimicrobial properties of HOCl and HOBr are well documented and numerous reports have provided strong evidence for severe damage to bacterial components, and bacterial proteins in particular, upon exposure to these oxidants within the neutrophil phagosome [215, 216]. Reaction of HOCl with neutrophil proteins as well as endogenous organic and inorganic amines in the phagosome lumen further leads to the formation of the longer-lived but less reactive chloramines monochloramine, N-chlorotaurine and protein derived chloramines with the latter being the predominant species due to the high abundance of proteins in the neutrophil phagosome [90, 215]. The formed chloramines have also been implicated in mediating cytotoxicity to a broad array of microorganisms [223].

HOCl and HOBr, once formed, readily react with a variety of functional groups on diverse biological molecules including proteins, DNA [11], cholesterol [224], and lipids [13]. HOCl and HOBr can target all cellular components. Nevertheless, proteins are likely to be the primary target for these oxidants, given their abundance in the cell and their high reactivity.

**Fig. 2:**
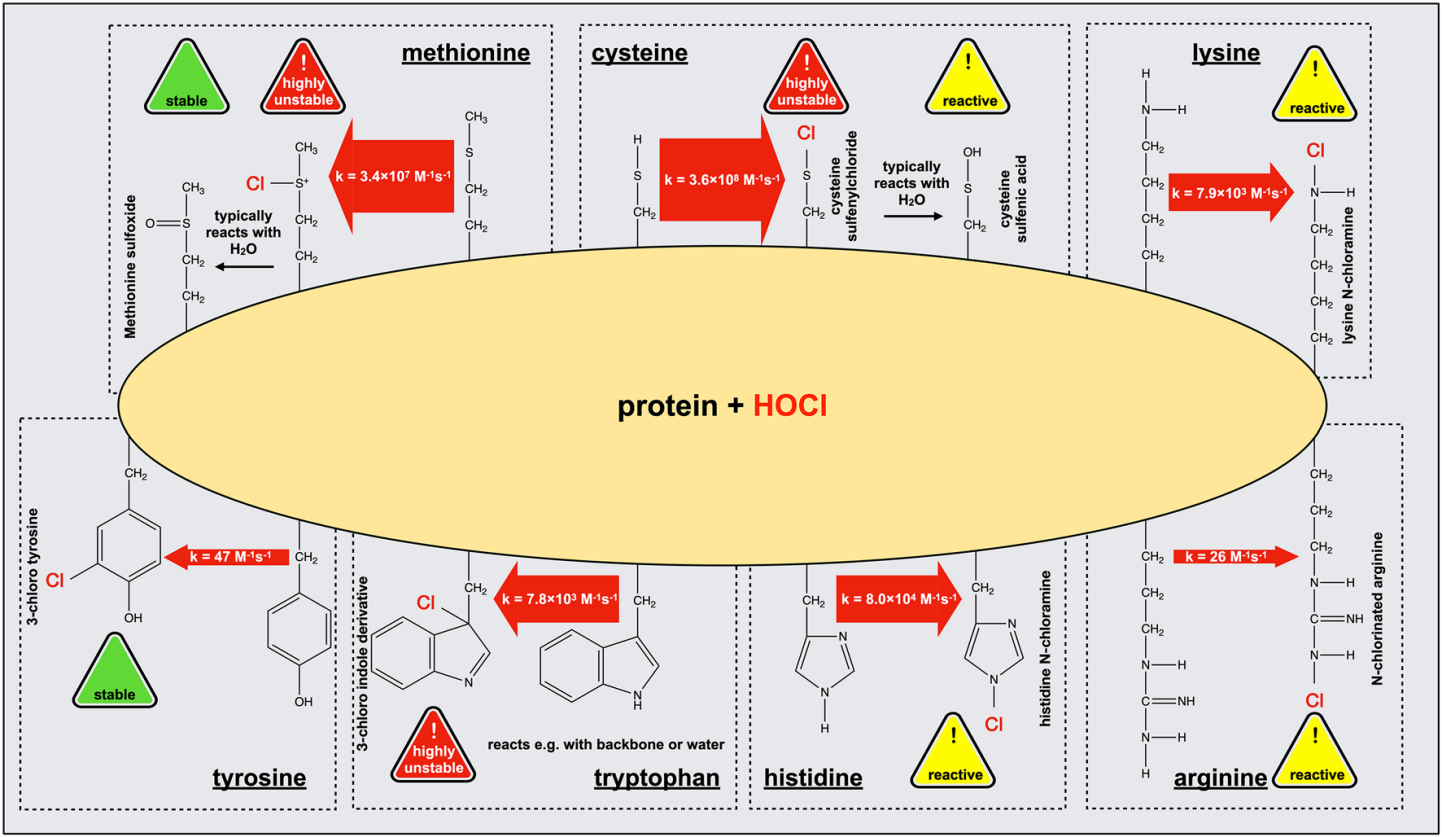
Reaction of HOCl with amino acid side chains in proteins. The initial reaction products of relevant amino acid side chains with HOCl are depicted. The width of the reaction arrows is proportional to the logarithm of the apparent second-order rate constant, a wider arrow thus indicates a faster reaction of the respective amino acid side chain by orders of magnitude. Rate constants obtained in experiments with model compounds as reported by the Davies group in refs [228, 229, 243]

Exposure of proteins to HOCl results in a broad range of modifications that have been very recently summarized in an excellent review by Hawkins [225] and only a short overview, based mostly on experimental data from the Davies and Hawkins groups, will be given here and in Fig. 2.

Reactivity of HOCl varies among the different amino acid side-chains, however, primary amines and sulfur-containing side-chains were found to be particularly prone to modification [226–228]. HOCl reacts rapidly with the sulfur-containing amino acids cysteine and methionine (with a second-order rate constant *k* = 3.6 × 10^8^ and *k* = 3.4 × 10^7^ M^−1^ s^−1^, respectively [229]). In comparison, HOBr oxidizes cysteine and methionine residues with a tenfold lower second-order rate constant *k* = 1 × 10^7^ M^−1^ s^−1^ and *k* = 3.6 × 10^6^ M^−1^ s^−1^, respectively [230].

Consistent with these overall rapid reaction rates of HOCl and HOBr with thiols, we found that exposure of phagocytized bacteria to oxidants produced during neutrophil respiratory burst leads to a rapid and massive breakdown of the thiol redox homeostasis of their proteome [179]. Cysteine thiols are presumably first chlorinated to form the unstable intermediate sulfenyl chloride, which rapidly reacts with water to yield a sulfenic acid (Fig. 2). This sulfenic acid is also highly unstable and can either react with a cysteine thiol group in close proximity to form a disulfide bond or become further oxidized to a sulfinic and sulfonic acid. Whereas sulfenic acid and disulfides can be reversed by antioxidant systems such as the thioredoxin (Trx) or glutaredoxin (Grx) systems [231, 232], sulfinic and sulfonic acid are generally considered irreversible modifications in prokaryotes which typically lead to inactivation and aggregation of proteins. Of note, sulfinic acids can, however, be reduced to thiols by sulfiredoxins in eukaryotic organisms as well as in cyanobacteria [233, 234].

Although less reactive (with second-order rate constants of *k* ≈ 1 × 10^4^–7 × 10^4^ M^−1^ s^−1^ [235]), HOSCN appears to be an even more thiol-specific oxidant than HOCl and HOBr [236]. HOSCN was found to selectively target cysteine residues in proteins in bacteria and host cells resulting in the formation of sulfenyl thiocyanate derivatives which can hydrolyze to sulfenic acid intermediates. Such sulfenyl species have indeed been reported in bacterial cells upon exposure to HOSCN [237]. Moreover, selenocysteine residues, that are typically present in the active site of protective antioxidant enzymes, such as glutathione peroxidase (GPx) and thioredoxin reductase (Trx), are also rapidly oxidized by HOSCN [238].

Unlike HOCl/HOBr, there are only limited experimental data available supporting the reactivity of HOSCN with biological targets other than protein thiols and selenols, and low-molecular-weight thiol compounds such as glutathione [235, 236, 238, 239]. Along this line, HOSCN has been reported to be much less effective in killing invading microorganism than HOCl/HOBr and is considered to be more cytostatic than cytotoxic in nature [240]. As HOSCN can rapidly penetrate bacterial membranes, potential targets of HOSCN include cytosolic thiol-dependent glycolytic enzymes, such as glyceralaldehyde-3-phosphate dehydrogenase (GAPDH), hexokinase, glucose-6-phosphate dehydrogenase, or aldolase. Oxidation of these metabolic enzymes results in interruption of the bacterial glycolytic flux and thus strongly decreases energy production reflected by severe growth inhibition [241]. Moreover, depletion of reduced glutathione by HOSCN could also lead to an increase in oxidative stress and bacterial susceptibility to neutrophil-generated oxidants [235, 236].

Reaction of HOCl with methionine results in the formation of stable methionine sulfoxides (Met(O)) [242]. Indeed, nearly 50% of all methionine residues in bacterial cytosolic and inner membrane proteins were found to be converted to Met(O) soon after phagocytosis. Methionine sulfoxidation can normally be reversed by the enzyme methionine sulfoxide reductase. *E. coli* strains lacking this enzyme consequently showed substantially increased sensitivity to HOCl providing evidence that methionine oxidation contributes to bacterial killing within neutrophil phagosomes [242].

Side-chain amines of lysine and arginine residues are the also targets in proteins for modification by HOCl (with a second-order rate constant *k* = 7.9 × 10^3^ M^−1^ s^−1^ for lysine and *k* = 26 M^−1^ s^−1^ for arginine) [228, 243]. Reaction of amines with HOCl leads to their chlorination (N-chlorination) to mono- and potentially dichloramines. N-chlorination is a reversible modification that can be fully removed by antioxidants such as ascorbate or, as we showed, the glutathione and Trx system, once the HOCl-stress has passed [15]. During the last decade, evidence has emerged that N-chlorination serves as a reversible switch to temporarily alter the function of bacterial and host proteins in response to HOCl [15, 37]. In the next chapters, we will discuss how N-chlorinated proteins can confer protection and contribute to bacterial survival within the neutrophil phagosome, and on the other hand, how they modulate host immune responses to accelerate pathogen clearance.

Reaction of the imidazole ring of histidine with HOCl (with a second-order rate constant *k* = 8.0 × 10^4^ M^−1^ s^−1^ [228]) leads to the formation of a short-lived chloramine. Aside from thiols and amines, HOCl also reacts with the indole moiety of tryptophan residues (with a second-order rate constant *k* = 7.8 × 10^3^ M^−1^ s^−1^ [228]) to form a 2-oxoindole derivative, although reactivity of tryptophan is two orders of magnitude higher with HOBr [230]. Finally, tyrosine residues were also found to be halogenated by HOCl and HOBr, yielding 3-chlorotyrosine (with a second-order rate constant *k* = 47 M^−1^ s^−1^ [178]) and 3-bromotyrosine, respectively. Although these halogenated molecules are minor products of the reaction of HOCl and HOBr with proteins, they are widely used as biomarkers to detect hypohalous acid-induced protein damage due to their specificity and high stability [215, 244].

In conclusion, exposure of proteins to HOCl and other hypohalous acids results in a wide range of oxidative modifications and formation of halogenated products. While most of those are considered detrimental to protein stability and result in fragmentation [245, 246], misfolding or cross-linking/aggregation [247], some others turned out to be beneficial under particular stress conditions (see next chapters).

## Microbial strategies to survive HOCl-stress

### Mechanisms of HOCl-mediated pathogen killing

There is little doubt that HOCl is of crucial importance for microbial killing in the neutrophil phagosome, but its mechanisms of action are still not fully understood [217]. Early studies noted already that HOCl promotes microbial death via several independent mechanisms by simultaneously acting on membranes [248, 249], proteins [250] and nucleotides [251].

HOCl was found to cause a rapid loss of glucose respiration and metabolic energy (1) by inhibiting proteins responsible for the transport of potential respiratory substrates such as glucose, succinate and amino acids across the inner membrane [252, 253] and (2) by inactivating membrane-localized F1-ATPase to disrupt bacterial ATP production [254]. Defective energy metabolism and loss of ATP inactivates essential ATP-dependent chaperone systems that normally assist in protein folding and prevent protein aggregation [255, 256]. As described in the previous chapter, one effective killing mechanism of HOCl may thus be the oxidative unfolding and irreversible aggregation of essential bacterial proteins [10]. This idea is supported by the observation that bacteria, which lack the HOCl-activated molecular chaperone Hsp33, accumulate a significant number of aggregated proteins and are much more sensitive to HOCl treatment than wild-type cells harboring functional Hsp33 [10]. Additionally, it was demonstrated that HOCl inhibits DNA replication in bacteria, which normally occurs in association with the inner membrane. Damage of inner membrane proteins involved in binding oriC, however, can result in a loss of this association and consequently, loss of DNA synthesis [257]. This combined data point toward cytosolic and inner membrane proteins as the primary target for HOCl attack.

### Bacterial strategies to maintain proteostasis during HOCl-stress

During evolution, bacteria evolved a diverse set of strategies to escape HOCl-inflicted damage and survive within the host environment. Bacterial responses to ROS-derived oxidative stress are well-characterized (reviewed in [258]) and growing evidence suggests that many of those are also involved in resisting HOCl-stress [259]. A comprehensive review of all bacterial defense mechanisms is beyond the scope of this article. Instead, as proteins are a major target of HOCl-mediated damage, we will briefly describe the general bacterial strategies to maintain a healthy proteome during redox imbalance and then highlight the role of redox-regulated chaperones which have been the subject of intensive research during the past years.

#### Increased production of antioxidants

One major principle by which bacteria protect themselves against HOCl is the expression of transcription factors and the upregulation of genes encoding antioxidant enzymes which confer resistance by detoxifying reactive oxygen and chlorine species and reducing amino acid side-chain modifications in proteins [260].

A broad range of different enzymes and non-enzymatic antioxidants act in concert to maintain a reducing environment in the cytoplasm. Given the high reactivity of HOCl with sulfur-containing cellular components, it is not surprising that bacteria possess several repair systems capable of repairing oxidatively damaged cysteine and methionine residues in the cytoplasm or membrane (reviewed in [259]). Reduction of oxidized cysteine residues to the thiol state in proteins is catalyzed by various oxidoreductases, including Trx and Grx, both of which were found to be upregulated under oxidative stress conditions. During reaction with disulfides in oxidized proteins, Trx and Grx become oxidized and subsequently reduced again by NADPH-dependent thioredoxin reductase or the low-molecular-weight thiol glutathione (GSH), respectively, to regenerate the active form of these enzymes. Glutathione is highly reactive with a variety of reactive oxygen and chlorine species, including HOCl, and thus is considered a crucial non-enzymatic antioxidant and scavenger of reactive oxygen and chlorine species in vivo [261]. In addition, GSH reacts with sulfenic acids in oxidized proteins forming glutathione–protein mixed disulfides and, as such, prevents their further oxidation to irreversible sulfonic and sulfinic acids [262]. Consistent with the important role of GSH in HOCl-stress resistance, *E.* *coli* mutants lacking GSH were found to be much more sensitive to HOCl and other chlorine species and generally more susceptible to neutrophil-mediated killing [263, 264].

During HOCl stress, methionine residues of bacterial proteins are oxidized into methionine sulfoxide (Met-S=O), leading to significant structural alterations, which might culminate in the loss of protein activity and function. Production of methionine sulfoxide reductase (Msr), which repairs such oxidized methionine residues in proteins, is thus upregulated in many bacteria such as *Bacillus* species, *Pseudomonas aeruginosa* and *E. coli* during HOCl-stress [265–268]. *E. coli* mutants deficient in Msr were found to be far more sensitive to HOCl, whereas overexpression of Msr led to higher HOCl resistance [242]. However, the ability of Msr to reverse the toxic effects of HOCl is only limited and depends on the HOCl amounts present [242]. Exposure of *E.* coli to 200 μM HOCl led to the oxidation of 40% of the cellular methionine residues and an almost complete loss of bacterial viability [242]. In the face of persistent oxidative stress and high HOCl levels, methionine sulfoxide can be further oxidized to methionine sulfone (Met-S-O_2_) [269], a modification that is no longer recoverable by Msr and, therefore, considered irreversible. Such irreversible methionine oxidation will permanently affect protein structure and function, thereby explaining the limited potential of Msr to counteract the toxic effects of HOCl.

#### Activation of ROS-sensing transcription factors

Aside from enzymatic and non-enzymatic antioxidants, growing evidence points toward the role of redox-sensitive transcription factors in protecting bacteria from the detrimental effects of HOCl. The first transcription factor in *E. coli* found to contribute to HOCl resistance was HypT (hypochlorite-responsive transcription factor, formerly known as YjiE) [270, 271]. HypT is activated by HOCl through oxidation of three methionine residues to methionine sulfoxide [271]. Once active, HypT was found to increase cell viability by upregulating genes involved in cysteine and methionine biosynthesis and sulfur metabolism to replenish oxidized metabolites, while repressing iron acquisition genes to limit the formation of highly toxic hydroxyl radicals through Fenton reaction [270, 271].

Another transcription factor that specifically responds to HOCl is NemR, which is activated via oxidation of HOCl-sensitive cysteine residues. NemR regulates expression of the enzymes glyoxalase and N-ethylmaleimide reductase, both of which are involved in detoxification of methylglyoxal and other reactive electrophiles [272].

Moreover, multiple studies in Gram-negative bacteria have shown that the ArcAB two-component signal transduction system, which normally acts as a global regulator of anaerobic growth of bacteria, also plays a role in the resistance to ROS and supports bacterial survival under oxidative stress conditions [273–275]. Although normally only active in the presence of low oxygen levels, exposure to the oxygen species H_2_O_2_ and HOCl also leads to its activation in several *Salmonella enterica* species, *Haemophilus influenzae* and other pathogens [276]. Upon activation, ArcA was found e.g. to modulate cellular metabolism and promote adaptation to changing oxygen levels or to downregulate abundant outer membrane porins that are responsible for the influx of these ROS by the phagocytized bacteria within the neutrophil phagosome [273, 274, 276].

#### Activation of novel chaperones

ATP-dependent molecular chaperones such as the well-studied GroEL (Hsp60) and GroES (Hsp10) system or the DnaK/DnaJ/GrpE (Hsp70/Hsp40) system assist in protein folding and play crucial roles in protecting proteins against oxidative stress-induced unfolding and aggregation [277–282]. Exposure to HOCl, however, causes a sudden and substantial drop in cellular ATP levels [252–254], rendering those chaperone systems devoid of their cofactor [256]. In addition, GroEL and DnaK, just like all other proteins, are critical targets for modification by HOCl, which can potentially lead to their inactivation [255, 256]. Hence, when protein unfolding occurs, cells can no longer rely on ATP-dependent foldases but require ATP-independent alternatives with a similar function instead to counteract the protein-damaging conditions during oxidative stress.

**Fig. 3:**
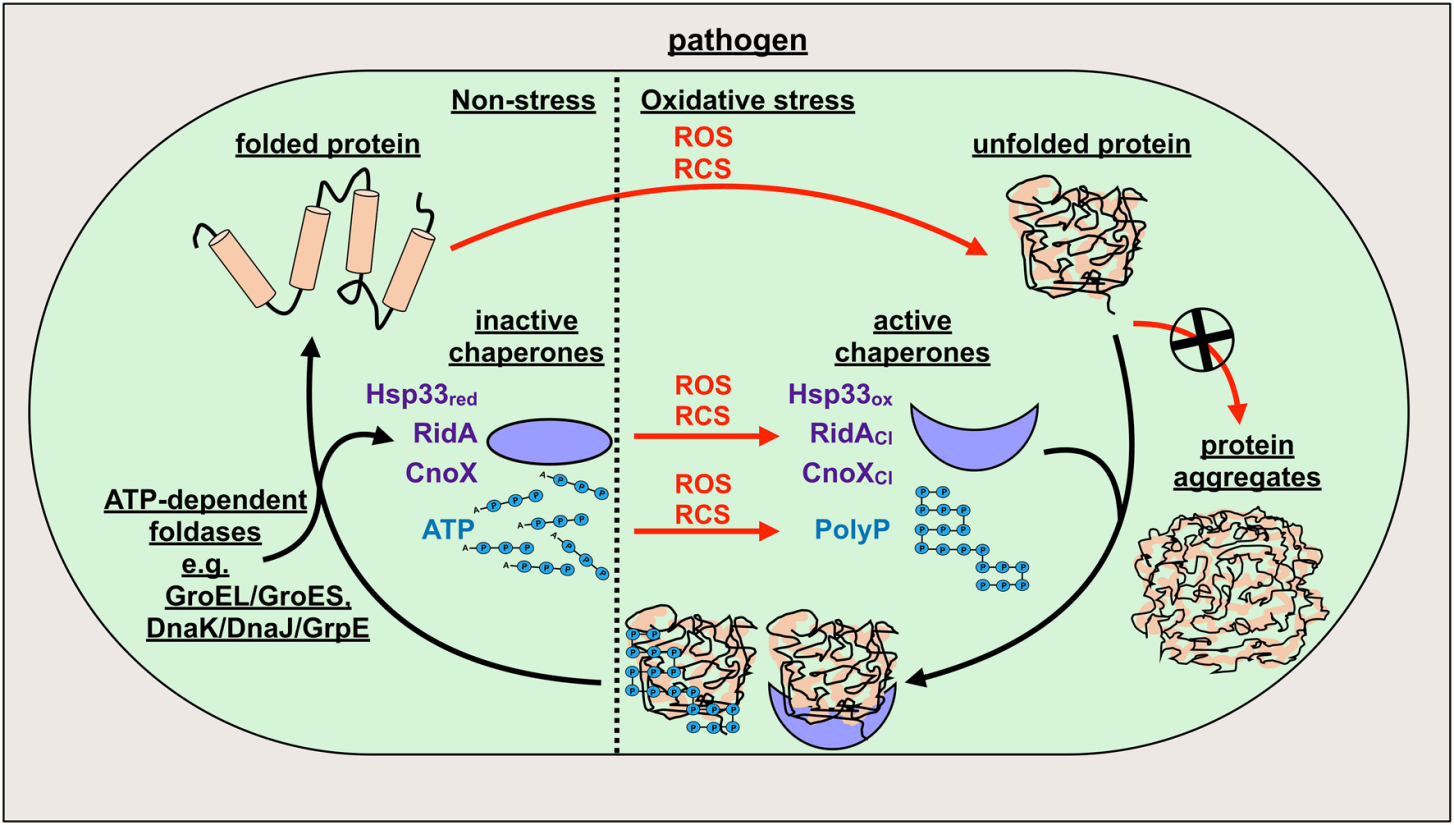
Stress-activated chaperone-like holdases protect bacterial proteins against aggregation. During oxidative stress, proteins become modified and oxidized by reactive oxygen and chlorine species (ROS/RCS), resulting in their unfolding and ultimately, aggregation. To prevent irreversible protein aggregation, the stress-induced ATP-independent holdases Hsp33 (Hsp33_red_), RidA and CnoX (violet) are activated during ROS/RCS-stress via oxidation (Hsp33_ox_) or chlorination (RidA_Cl_, CnoX_Cl_), allowing them to bind and protect other unfolding proteins. Moreover, cellular ATP (blue) is converted to inorganic polyphosphates (polyP), which by themselves can act as ATP-independent chaperones, effective in stabilizing unfolding proteins and preventing protein aggregation. Once the stress subsides and cellular ATP levels are restored, the stress-induced holdases are reduced or, in the case of polyP, disassembled and could pass their substrates to ATP-dependent foldases such as GroEL/GroES and DnaK/DnaJ/GrpE for proper refolding

Recent studies in *E. coli* revealed that at least some loss of ATP upon HOCl stress is due to conversion of cellular ATP to inorganic polyphosphates (polyP), which by themselves can act like ATP-independent chaperone holdases, effective in stabilizing unfolding proteins and preventing protein aggregation both in vivo and in vitro [282, 283] (Fig. 3).

Over the last decade, several accessory chaperones have been identified which are specifically activated in response to oxidative stress (recently reviewed in [284]). Activation of their chaperone function occurs post-translationally through oxidation of redox-sensitive cysteine residues (e.g. Hsp33 or 2-Cys-peroxiredoxins in prokaryotes and Get3 in eukaryotes) or chlorination of side-chain amines (e.g. RidA and CnoX in *E. coli*) by ROS, HOCl or other chlorine species (Fig. 3). These novel chaperones are ATP-independent holdases, which bind to and protect unfolding proteins from aggregation but do not promote their refolding [284]. When oxidative stress has passed and cellular ATP levels are restored, the stress-induced holdases could transfer their substrates to ATP-dependent chaperone systems, such as DnaK/DnaJ/GrpE or GroEL/GroES, for proper folding.

Hsp33 was the first redox-regulated chaperone identified [10, 284, 285]. While normally inactive, Hsp33 becomes transiently transformed into an efficient holdase-type chaperone when oxidative and unfolding conditions coincide. Activation of Hsp33’s chaperone functions relies on two stress sensors, a redox sensitive zinc center and a thermolabile region, both located in the C terminus of the protein [286]. Under reducing, non-stress conditions, four highly conserved cysteine residues, which constitute one of the stress sensor domains, are kept in their reduced thiol state and together coordinate one zinc(II) ion [287]. Upon exposure to HOCl, these cysteine thiols are oxidized to form two disulfide bonds, releasing the zinc ion. This redox event is accompanied by major structural rearrangements and unfolding of the protein’s second stress sensor region, leading to the dimerization of Hsp33, a crucial step to fully activate Hsp33’s chaperone function [10, 285, 286]. Active Hsp33 is capable of recognizing and binding unfolding cellular proteins and thus prevents irreversible protein aggregation as long as stress conditions persist. Since Hsp33’s chaperone function is not driven by ATP, Hsp33 can adequately compensate for the loss of ATP-dependent chaperone systems. Importantly, Hsp33 returns to its original, chaperone-inactive state once the HOCl-stress subsides, making its sensor domains reversible and transient functional switches. Interestingly, the structurally unrelated eukaryotic protein Get3 has been recently found to also function as ATP-independent chaperone-like holdase during oxidative stress. Get3 senses oxidants with a zinc center, similar to prokaryotic Hsp33 [288, 289].

While redox-mediated activation of Hsp33’s and Get3’s chaperone function is initiated by oxidation of specific “stress-sensing” cysteine residues, other proteins, such as *E. coli* RidA and CnoX, were found to be converted into efficient chaperone holdases via a distinct activation mechanism that specifically involves chlorination of their side-chain amines [15, 16].

The *E. coli* protein RidA, a member of the highly conserved, but functionally highly diverse YjgF/YER057c/UK114 protein family, acts as an enamine/imine deaminase that detoxifies reactive intermediates generated during the course of amino acid metabolism [290]. We discovered that RidA functions as a highly efficient and ATP-independent chaperone holdase under HOCl-stress conditions [15]. Activation of RidA’s chaperone function occurred only in the presence of reactive chlorine species, such as HOCl, while exposure to H_2_O_2_ and other oxidants had no effect. This functional conversion of RidA in response to HOCl did not depend on cysteine oxidation since a cysteine-free RidA variant showed similar chaperone activity upon HOCl exposure. Moreover, treatment with HOCl substantially decreased RidA’s levels of free amino groups and led to an overall increase in surface hydrophobicity and formation of higher oligomers, which can also be observed with other stress-activated chaperones. Finally, exposure of HOCl-treated RidA to antioxidants fully abolished its chaperone activity. These combined observations prompted us to conclude that reversible N-chlorination of lysine and/or arginine side-chains is likely responsible for the activation of RidA’s chaperone function. N-chlorination thus serves as an alternative, cysteine-independent mechanism to employ novel chaperones in response to HOCl. Since RidA’s chaperone function is not dependent on ATP, RidA, similar to Hsp33, is perfectly suited to function under HOCl-stress conditions, which transiently incapacitate ATP-dependent chaperone systems.

While activation of chaperone function by thiol oxidation typically requires the oxidation of particular cysteine residues, activation by N-chlorination appears to be rather unspecific and likely due to a general increase in surface hydrophobicity. Hence, many more proteins might undergo similar HOCl-triggered conversion into effective chaperone holdases than assumed, building up a protective shield against HOCl-induced protein aggregation. Support for this notion has been very recently provided by Goemans and colleagues who found that *E. coli* CnoX also turns into a powerful chaperone holdase by N-chlorination in a mechanism similar to that of RidA activation [16]. Under HOCl-stress conditions, CnoX does not only act as a holdase but forms mixed-disulfide complexes with its substrates and thus prevents redox-sensitive cysteine residues from being irreversibly oxidized. Due to this dual function, Goemans et al. described CnoX as the first member of a new class of proteins, the so-called “chaperedoxins” [16]. Of note, CnoX transfers its client proteins to both GroEL/GroES and DnaK/DnaJ/GrpE chaperone system for refolding once the HOCl-stress has passed. Whether RidA is also capable of interacting with these chaperone foldase systems needs to be elucidated.

Since the absence of any of these chaperones renders the bacteria sensitive to HOCl [15, 16], protein unfolding and aggregation is apparently one major mechanism by which HOCl contributes to microbial cell death.

We recently discovered that many human plasma proteins are also specifically converted into chaperone-like holdases by N-chlorination and as such, gain the ability to protect other proteins from HOCl-induced aggregation [37]. In the following chapters, we will briefly discuss the effects of HOCl and other hypohalous acids on the host in general and then focus on the current understanding of how HOCl modulates the function of host proteins during infection and inflammation.

## Effects of HOCl on the host environment

### Role of extracellular MPO in host tissue damage

There is little doubt that MPO-generated hypohalous acids, particularly HOCl, are of crucial importance for microbial killing. Excessive or inappropriate formation of these oxidizing agents, however, can potentially lead to undesirable damage of host cells through the same processes used in the destruction of invading pathogens [291–294].

Numerous studies have provided strong evidence for the extracellular presence of enzymatically active MPO at sites of infection and inflammation. This localization may arise either from MPO secretion into phagolysosomes followed by disruption of this compartment (e.g. due to cell lysis), attachment of MPO to extracellular NETs or via the erroneous release of MPO at the plasma membrane as a result of inappropriate intracellular trafficking [295]. Irrespective of the exact mechanism responsible for extracellular MPO release, there is little doubt that this enzyme can induce extensive damage to host tissue due to persistent generation of hypohalous acids [205].

However, as the levels of MPO itself as well as the availability of appropriate substrates can vary widely within the organism and the particular sites of inflammation, the extent of damage generated by this enzyme often cannot be accurately predicted. Compared to the intracellular phagosomal space where the substrate availability is controlled, the MPO-dependent oxidative biochemistry in the extracellular environment is usually far more diverse, leading to substantial modifications of a wide variety of biomolecules, including DNA, lipids, carbohydrates and proteins. Accordingly, increased levels of MPO and excessive generation of its associated oxidants, particularly HOCl, have been causally linked to the development of several types of major inflammatory pathologies such as atherosclerosis, neurodegenerative disorders, rheumatoid arthritis, lung diseases, kidney diseases, diabetes and cancer [21]. While the specific effects of HOCl on host tissues have already been studied intensely, there is only limited experimental data available on the role of other hypohalous acids, e.g. HOSCN, in the pathogenesis of those diseases. Unlike HOCl and HOBr, which, in part, appear to function as membrane-lytic oxidants, HOSCN has only restricted reactivity and thus can easily penetrate bacterial and mammalian cells, leaving their membranes intact [296–298]. Despite its beneficial role in host defense against invading pathogens, the high selectivity of HOSCN for intracellular thiols and thiol-dependent enzymes may be detrimental to host tissue as well, particularly under chronic inflammatory conditions. Hence, there is emerging evidence that depletion of the major antioxidant GSH and reversible inactivation of key thiol-dependent enzymes, including protein tyrosine phosphatases (PTPs), creatine kinase (CK), GAPDH, glutathione S-transferases (GSTs), and various membrane ATPases, can potentially elicit a response similar to that observed in bacterial cells, resulting in cellular dysfunction and cell death [239, 299–302]. However, the ability of HOSCN to effectively induce mammalian cell damage and contribute to disease pathogenesis is still controversial. Certain mammalian cell types, including erythrocytes, macrophages and endothelial cells, are highly susceptible to the actions of this oxidant, while other cell types, particularly those associated with the respiratory tract [303, 304] or the oral cavity [305, 306], seem to be rather HOSCN resistant. Similarly, elevated plasma levels of HOSCN, for example in smokers, together with the ability of SCN^−^ to scavenge other oxidants, including HOCl and HOBr, have been proposed to provide protection against tissue injury and inflammation due to the shifted ratio of MPO-derived halogenating species and thus decreased amounts of the more damaging inflammatory agents HOCl and HOBr [212, 307, 308].

Normal MPO concentration in human plasma ranges from 18 to 39 ng/ml [309, 310] and was found to be significantly elevated to averages of 55 ng/ml [310] and 287 ng/ml [311] after myocardial infarction and acute coronary syndrome, respectively. Thus, enhanced levels of MPO activity are widely considered a useful oxidative stress biomarker and diagnostic tool for many of those commonly-occurring inflammatory diseases.

Apart from the strong oxidizing properties of its associated oxidants, MPO, as a strongly basic protein, can also bind to the negatively charged glycocalyx on the surface of several cell types, such as epithelial and endothelial cells [312, 313], macrophages, or neutrophils [313]. By reducing the anionic surface charge of the endothelial glycocalyx, MPO facilitates neutrophil recruitment to sites of infection/inflammation, independent of its classic catalytic function [313]. Aside from this electrostatic effect, MPO also acts as extracellular signaling molecule and modulator of immune cell activation. For instance, the interaction of MPO with neutrophil CD11b/CD18 integrins enhances tyrosine phosphorylation, leading to the activation of protein tyrosine kinases that are involved in the regulation of degranulation and neutrophil respiratory burst [314]. Both, the enhanced influx of neutrophils from blood to the inflammatory site and their increased stimulation can drastically intensify inflammation, thus supporting the role of extracellular MPO in the propagation of inflammatory pathologies.

Neutrophils that accumulate in the interstitial fluid of inflamed tissues have been reported to produce HOCl at concentrations of up to 25–50 mM/h [315]. However, the extracellular concentration of this oxidant is difficult to predict, as it does not only depend on the amount of neutrophils in the inflammatory region, but also on the levels of MPO released during neutrophil activation and availability of H_2_O_2_. HOCl, once generated, typically does not accumulate to high levels, as it reacts rapidly with various biological molecules present in its immediate vicinity [11, 13, 29, 224, 243]. Due to high abundance in blood and interstitial fluids, HSA and other plasma proteins are thought to be the major target of HOCl-mediated damage [24–28]. Treatment of plasma with HOCl led to the rapid depletion of thiol groups and methionine residues in the proteins, demonstrating the potential of plasma proteins, particularly HSA, to effectively scavenge HOCl [316]. Although oxidants, such as ascorbate, also react rapidly with HOCl, their plasma concentrations are too low when compared to protein thiols and methionines for them to act as major HOCl scavengers [317].

Reaction of HOCl with normal tissue and plasma proteins causes the formation of so-called “advanced oxidation protein products (AOPPs)” [23, 32]. Accumulation of such AOPPs has been first reported in patients with chronic kidney disease [23] and was later also found in several other inflammatory diseases, such as rheumatoid arthritis, cardiovascular disease, and neurodegenerative disorders (reviewed in [21]).

In the last two decades, a number of studies have been carried out to elucidate the role of HOCl-modified plasma proteins in inflammatory processes [22, 33, 36, 318]. Intriguingly, while in some cases exposure to HOCl had disastrous effects on the structure or function of a protein leading to its aggregation and inactivation, other proteins were found to undergo a functional switch upon modification by HOCl that may be beneficial for the host defense against pathogens but may also contribute to chronic inflammation.

### Effect of HOCl-induced modifications on the function of plasma proteins

Pathogenesis of a number of inflammatory diseases and tissue injuries is associated with modification and/or inactivation of host proteins by extracellular HOCl.

One prominent effect of HOCl at inflammatory sites is the inactivation of protease inhibitors. In vitro studies revealed that HOCl modifies and inactivates α_1_-antiproteinase and α_2_-macroglobulin, both of which are known to inhibit several proteolytic enzymes released from microbes and neutrophil granules in extracellular fluids [153, 319, 320]. Uncontrolled extracellular activity of proteases, such as elastase, may inadvertently damage host tissues for example by breaking down connective tissue fibers, such as elastin in the lung [321]. However, whether these antiproteases are significantly targeted by HOCl in vivo is not clear, since HOCl is readily scavenged by antioxidants, HSA and other plasma proteins [29].

Ceruloplasmin, an abundant acute phase protein in plasma, acts as an important antioxidant, which has been shown to directly bind and inhibit MPO [322], interfere with HOCl production [323] and thus, for instance, prevent HOCl-mediated degradation of the α_1_-antiproteinase and other proteins [30]. Recently, it has been reported that ceruloplasmin is also modified by HOCl during inflammation [324]. Reaction of ceruloplasmin with HOCl leads to its denaturation and the formation of large aggregates [325]. Importantly, ceruloplasmin appears to lose its ability to inhibit MPO upon modification by HOCl [324]. Aside from ceruloplasmin, complement C3 was also found to associate with MPO in plasma [322, 323].

Modification of low-density lipoprotein (LDL) by HOCl has been implicated in human atherosclerosis. Exposure of LDL to HOCl leads to its aggregation, followed by rapid uptake and degradation by macrophages [247, 326, 327]. The unregulated uptake of oxidized LDL is considered a crucial step in the conversion of macrophages into foam cells [328]. Furthermore, N-chloramines from HOCl-modified LDL have been reported to inactivate lecithin-cholesterol acyltransferase (LCAT), an enzyme involved in the maturation of the antiatherogenic high-density lipoprotein (HDL), thus providing another mechanism by which HOCl promotes atherogenesis [329].

Interestingly, HOCl-oxidized LDL, as much as HOCl-modified HSA, have been shown to trigger various neutrophil responses such as activation of the NADPH oxidase, degranulation or shape change [32, 37, 38, 330] but until recently, the mechanism underlying this HOCl-mediated functional conversion has been unclear. In general, pathologically elevated concentrations of HOCl, as those present in chronically inflamed tissues, can induce a wide variety of modifications on plasma proteins such as carbonylation, cysteine and methionine oxidation, N-chlorination or di-tyrosine crosslinking [156, 330–332], most of which are considered irreversible.

**Fig. 4:**
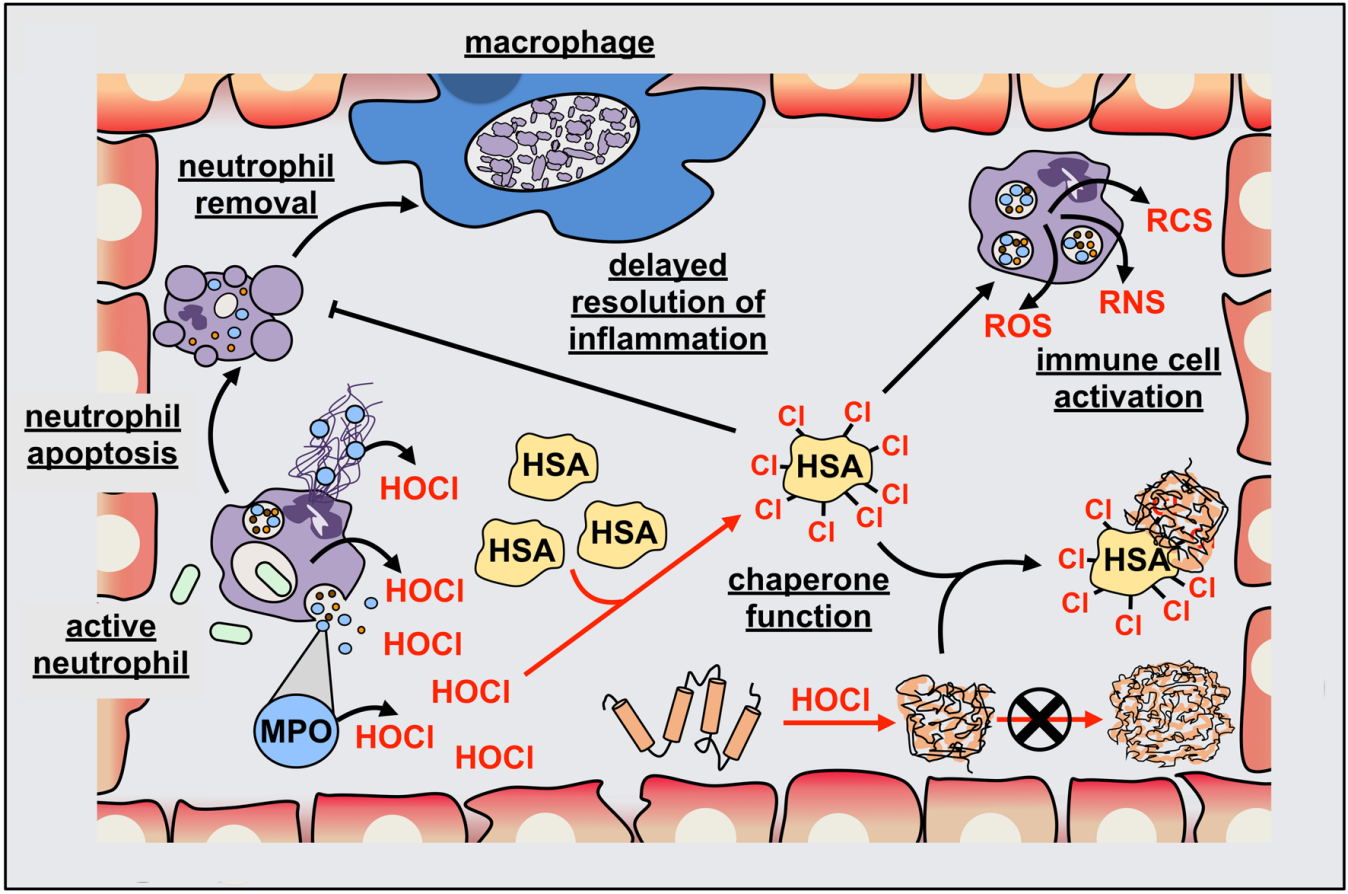
Immunomodulatory role of HOCl-modified human serum albumin (HSA) during infection and inflammation. At the site of infection or inflammation, activated neutrophils release myeloperoxidase (MPO; blue circle) into the extracellular surroundings via leakage during phagocytosis, degranulation or association with neutrophil extracellular traps (NETs). Extracellular MPO generates hypohalous acids, particularly hypochlorous acid (HOCl), in the immediate vicinity to host cells and host proteins, causing oxidative damage. Due to its high abundance in blood and interstitial fluid, HSA (yellow) is considered a major scavenger of HOCl and as such, becomes modified upon HOCl-stress. Reversible chlorination of its basic amino acid side-chains (N-chlorination) by HOCl results in the activation of HSA’s chaperone function, allowing HSA to effectively bind and protect other proteins from HOCl-induced aggregation. Moreover, N-chlorination also turns HSA into a potent activator of immune cells and thereby increases the generation of reactive oxygen, nitrogen or chlorine species (ROS/RNS/RCS) by these cells. Finally, HOCl-modified HSA can extend neutrophil lifespan at sites of infection and inflammation by inhibiting cell apoptosis and thus delays the removal of neutrophils by macrophages and the resolution of inflammation. Both, the persistent activation of neutrophils and neutrophil lifespan extension provide a potentially detrimental positive feedback loop that may ultimately lead to chronic inflammation

We discovered that HSA, IgG and the majority of other human plasma proteins are transformed into potent inducers of the neutrophil respiratory burst specifically by N-chlorination [37], the same mechanism by which bacteria were found to employ novel chaperones to protect their proteins against HOCl-stress-induced aggregation [15, 16] (Fig. 4). Furthermore, HOCl-modified HSA was shown to enhance the survival of neutrophils in the presence of highly immunogenic foreign antigens by binding to and preventing their uptake by the immune cells [37]. The potential of HOCl-modified HSA to bind proteins secreted by pathogens has also been reported before by others [333, 334]. HOCl-modified HSA can thus be considered a pro-inflammatory mediator, that together with other inflammatory stimuli, such as cytokines, PAMPs or DAMPs, extends neutrophil lifespan at sites of tissue injury and inflammation through the inhibition of cell apoptosis [45–47] (Fig. 4).

Aside from their role in modulation of the innate immune response, we also found that HOCl-modified plasma proteins act as highly effective holdase-like chaperones, protecting other proteins from HOCl-induced aggregation [37] (Fig. 4). Wyatt et al. demonstrated that exposure of the extracellular chaperone α_2_-macroglobulin to HOCl improves its chaperone function [39]. We showed that not only α_2_-macroglobulin’s efficiency is enhanced, but a wide range of plasma proteins can be converted into chaperone-like holdases upon modification by HOCl [37]. As mentioned in the previous chapters, N-chlorination is one mechanism by which bacterial proteins are transformed into ATP-independent chaperone holdases in response to HOCl stress [15, 16]. It was thus little surprise that the functional switch of human plasma proteins turned out to be mediated by N-chlorination as well [37].

These combined findings demonstrate that HOCl-mediated N-chlorination may constitute a key mechanism to confer protection against HOCl-mediated protein aggregation found in both bacteria and humans. Furthermore, there is more and more evidence for a crucial role of N-chlorination in the modulation of host protein function to amplify and sustain host immune responses to infection and inflammation. Although the increased generation of NADPH oxidase-derived oxidants by neutrophils mediated by HOCl-modified plasma proteins can accelerate pathogen clearance at the site of an acute infection, prolonged lifespan of neutrophils due to inhibited apoptosis can also delay the resolution of inflammation, thus providing a positive feedback loop that may ultimately lead to chronic inflammation. It, therefore, appears likely that HOCl contributes to the pathogenesis of a number of inflammatory diseases through the modification of plasma proteins. Since plasma protein-derived chloramines are much more stable and longer lasting than HOCl, they can diffuse greater distances and potentially exert their effects also at remote sites within the human body [29, 153]. However, due to the reversible nature of N-chlorination, N-chloramines can be readily reduced and detoxified by antioxidants in the blood, providing an off-switch for this potentially detrimental feedback loop.

### The role of MPO and HOCl as a potential internal signaling molecule

After we have looked at the effects of neutrophil-generated hypohalous acids on phagocytized bacteria and the host tissue, we now want to briefly turn our attention to potential internal effects on the producing cells themselves. Using genetically encoded redox probes based on roGFP2, we could recently show that the cytosolic thiol redox homeostasis in neutrophil-like cells shifts to a more oxidized state upon activation [132]. This activation could be triggered by bacterial phagocytosis as well as the addition of PMA to the cells. Activation did not lead to full, but a more gradual oxidation of the probe. Additionally, inhibition of MPO did not change the oxidation of the probe. In light of the fact that HOCl is a highly effective thiol oxidant (it is, after all, the main factor in the oxidation of thiols in phagocytized bacteria [179] and leads to a full collapse of the bacterial thiol redox homeostasis [335]), we concluded that neutrophils must have a highly effective defense that prevents HOCl or other reactive chlorine species from leaving the confines of the phagolysosome, at least in amounts that could affect the overall thiol redox state of the neutrophil’s cytosol.

Nevertheless, it could be argued that HOCl, given its overall abundance in neutrophils could also play a role as a signaling molecule, especially in activated neutrophils such as those actively engaged in phagocytosis. Redox signaling, in which the ROS H_2_O_2_ acts as the signaling molecule, is a well-established concept (see [336, 337] for in-depth reviews). Similarly, there are first examples for HOCl-based signaling: the extracellular action of HOCl in the induction of apoptosis in transformed cells has been termed the HOCl-signaling pathway (see [338] for a recent review). This pathway relies on the extracellular reaction of HOCl with superoxide to generate the apoptosis-inducing hydroxyl radical [339], however, this reaction might be limited by the low abundance of both superoxide and HOCl in vivo. The presence of HOCl can also affect the phosphorylation state of protein kinase Cθ leading to insulin resistance, however, this signaling is mediated via peroxynitrite [340]. The indirect nature of these examples indicates that, due to its high reactivity with biomolecules, a direct HOCl-mediated signaling, if it happens at all, would need to occur in close spatiotemporal vicinity to the site of its generation. This would be especially true within the cytosol and its abundance of thiols, as HOCl is able to react with those at exceptionally high rates of more than 10^8^ M^−1^ s^−1^ [229].

Still, a number of examples for regulatory effects have been found: HOCl influences iron metabolism [341], stimulates the mitogen-activated protein (MAP) kinase pathway [342], induces translocation of transcription factors into the nucleus of T-lymphocytes [334], regulates cell growth by activating tumor suppressor proteins [343], or controls the enzyme activity of metalloproteinases [344]. Also, MPO was observed to modulate the vascular signaling and vasodilatory functions of nitric oxide (NO·) during acute inflammation by regulating NO· bioavailability [345]. There is also clear experimental evidence that HOCl-production is necessary to effectively induce NETosis in neutrophils, hinting at a role as messenger. Metzler et al. found that neutrophils of patients lacking MPO are defective in NET formation as well [181]. Low levels of MPO-derived hypochlorous acid have been reported to regulate NET release by neutrophils [346]. Further studies showed that it is the endogenous HOCl that facilitates NET formation, as extracellular addition of MPO did not rescue MPO-deficient neutrophils stimulated with PMA [347]. Additionally, we showed, that while the increase in overall thiol oxidation in neutrophil-like cells engaged in phagocytosis is not dependent on MPO, HOCl-production was still crucial for subsequent NET formation [132]. However, there are still major gaps in our understanding of the involvement of HOCl in the neutrophil’s internal signaling. Two major pathways are involved in both NET formation and ROS production in activated neutrophils. One is dependent on phosphoinositide 3-kinase (PI3K), the other on protein kinase C (PKC). Using inhibitors of these pathways, it was shown that the PKC-dependent pathway is necessary for neutrophils activated with PMA, whereas in neutrophils phagocytosing *E.* *coli*, the inhibition of PI3K had a larger effect. Nevertheless, in both cases inhibition of MPO lead to lower NET production [132]. This suggests that HOCl could either interact with both of these pathways or affect other pathways as well. In the particular case of NET production, it has been suggested that a rather unspecific peroxidation of membrane lipids could simply enable the translocation of granular proteins into the nucleus, thereby facilitating NETosis [347]. Others have suggested that the presence of the MPO protein, but not necessarily its activity, is required to promote NETosis [140, 348], however, the aforementioned studies showing effects of MPO inhibitors seem to suggest that the mere presence of the enzyme itself is not sufficient. Overall, HOCl might as well be a non-specific activator of several kinase pathways and, taking into account the fact that protein tyrosine phosphatases and dual specificity phosphatases have a conserved thiol in their active site, it is very likely that HOCl has a profound impact on the phosphoproteome and thus on regulatory pathways in the host.

## Conclusions

Neutrophils employ different strategies to kill off pathogens. Among the most effective weapons in their arsenal are hypohalous acids, such as HOCl. These strong oxidants can damage virtually any biomolecule, making them highly effective antimicrobials. However, recent research highlighted that particularly HOCl-induced protein modifications do not exclusively have damaging effects but can activate chaperone-like holdase functions in some proteins through thiol oxidation and N-chlorination. In the model bacterium *Escherichia coli* at least three proteins have been found that, once modified by HOCl, can prevent HOCl-induced protein aggregation: Hsp33, RidA and CnoX. Genetic experiments showed that the presence of these proteins protects *E. coli* from HOCl stress. But not only bacteria experience HOCl-stress in their encounter with immune cells. Hypohalous acids generated by immune cells either directly or through the release of MPO can also damage host molecules. We found that serum proteins are modified by reversible N-chlorination at concentrations of HOCl as they occur in the direct vicinity of inflammation. The majority of thiol groups in serum proteins are engaged in structural disulfides, and, using model proteins, it has been shown that these can react with hypohalous acids, at apparent second-order rate constants of up to 2.5 × 10^7^ M^−1^ s^−1^ [349]. Nevertheless, N-chlorination of basic amino acid side-chains is probably one of the predominant protein modifications induced by HOCl in serum proteins. These N-chlorinated serum proteins can then bind aggregating proteins and prevent their precipitation, activate immune cells, and protect them from cytotoxic antigens. Furthermore, hypohalous acids seem to be critically involved in the modulation of diverse signaling pathways in the cells that produce them, the neutrophils. Unfortunately, much of our knowledge about the effects of HOCl is derived from biochemical in vitro experiments. Future experiments will have to elucidate the physiological relevance of these findings and need to establish in vivo evidence of HOCl-mediated signaling events.

## References

[1] Yang P, Li Y, Xie Y, Liu Y (2019). Different faces for different places: heterogeneity of neutrophil phenotype and function. J Immunol Res.

[2] Papayannopoulos V (2018). Neutrophil extracellular traps in immunity and disease. Nat Rev Immunol.

[3] Mortaz E, Alipoor SD, Adcock IM, Mumby S, Koenderman L (2018). Update on neutrophil function in severe inflammation. Front Immunol.

[4] Nicolás-Ávila JÁ, Adrover JM, Hidalgo A (2017). Neutrophils in Homeostasis, Immunity, and Cancer. Immunity.

[5] Rosales C (2018). Neutrophil: a cell with many roles in inflammation or several cell types?. Front Physiol.

[6] Chapman ALP, Skaff O, Senthilmohan R, Kettle AJ, Davies MJ (2009). Hypobromous acid and bromamine production by neutrophils and modulation by superoxide. Biochem J.

[7] Furtmüller PG, Burner U, Obinger C (1998). Reaction of myeloperoxidase compound I with chloride, bromide, iodide, and thiocyanate. Biochemistry.

[8] van Dalen CJ, Whitehouse MW, Winterbourn CC, Kettle AJ (1997). Thiocyanate and chloride as competing substrates for myeloperoxidase. Biochem J.

[9] Winterbourn CC (2008). Reconciling the chemistry and biology of reactive oxygen species. Nat Chem Biol.

[10] Winter J, Ilbert M, Graf PCF, Ozcelik D, Jakob U (2008). Bleach activates a redox-regulated chaperone by oxidative protein unfolding. Cell.

[11] Prütz WA (1996). Hypochlorous acid interactions with thiols, nucleotides, DNA, and other biological substrates. Arch Biochem Biophys.

[12] Henderson JP, Byun J, Mueller DM, Heinecke JW (2001). The eosinophil peroxidase-hydrogen peroxide-bromide system of human eosinophils generates 5-bromouracil, a mutagenic thymine analogue. Biochemistry.

[13] Winterbourn CC, van den Berg JJ, Roitman E, Kuypers FA (1992). Chlorohydrin formation from unsaturated fatty acids reacted with hypochlorous acid. Arch Biochem Biophys.

[14] Schramm FD, Schroeder K, Jonas K (2019). Protein aggregation in bacteria. FEMS Microbiol Rev.

[15] Müller A, Langklotz S, Lupilova N, Kuhlmann K, Bandow JE, Leichert LIO (2014). Activation of RidA chaperone function by N-chlorination. Nat Commun.

[16] Goemans CV, Vertommen D, Agrebi R, Collet J-F (2018). CnoX is a chaperedoxin: a holdase that protects its substrates from irreversible oxidation. Mol Cell.

[17] Davies MJ, Hawkins CL (2020). The role of myeloperoxidase in biomolecule modification, chronic inflammation, and disease. Antioxid Redox Signal.

[18] Pravalika K, Sarmah D, Kaur H, Wanve M, Saraf J, Kalia K, Borah A, Yavagal DR, Dave KR, Bhattacharya P (2018). Myeloperoxidase and neurological disorder: a crosstalk. ACS Chem Neurosci.

[19] Ndrepepa G (2019). Myeloperoxidase—a bridge linking inflammation and oxidative stress with cardiovascular disease. Clin Chim Acta.

[20] Kisic B, Miric D, Dragojevic I, Rasic J, Popovic L (2016). Role of myeloperoxidase in patients with chronic kidney disease. Oxid Med Cell Longev.

[21] Aratani Y (2018). Myeloperoxidase: its role for host defense, inflammation, and neutrophil function. Arch Biochem Biophys.

[22] Descamps-Latscha B, Witko-Sarsat V, Nguyen-Khoa T, Nguyen A-T, Gausson V, Mothu N, London GM, Jungers P (2005). Advanced oxidation protein products as risk factors for atherosclerotic cardiovascular events in nondiabetic predialysis patients. Am J Kidney Dis.

[23] Witko-Sarsat V, Friedlander M, Capeillère-Blandin C, Nguyen-Khoa T, Nguyen AT, Zingraff J, Jungers P, Descamps-Latscha B (1996). Advanced oxidation protein products as a novel marker of oxidative stress in uremia. Kidney Int.

[24] Shao B, Oda MN, Bergt C, Fu X, Green PS, Brot N, Oram JF, Heinecke JW (2006). Myeloperoxidase impairs ABCA1-dependent cholesterol efflux through methionine oxidation and site-specific tyrosine chlorination of apolipoprotein A-I. J Biol Chem.

[25] Peng D-Q, Wu Z, Brubaker G, Zheng L, Settle M, Gross E, Kinter M, Hazen SL, Smith JD (2005). Tyrosine modification is not required for myeloperoxidase-induced loss of apolipoprotein A-I functional activities. J Biol Chem.

[26] Colombo G, Clerici M, Altomare A, Rusconi F, Giustarini D, Portinaro N, Garavaglia ML, Rossi R, Dalle-Donne I, Milzani A (2017). Thiol oxidation and di-tyrosine formation in human plasma proteins induced by inflammatory concentrations of hypochlorous acid. J Proteomics.

[27] Himmelfarb J, McMonagle E (2001). Albumin is the major plasma protein target of oxidant stress in uremia. Kidney Int.

[28] Fogh-Andersen N, Altura BM, Altura BT, Siggaard-Andersen O (1995). Composition of interstitial fluid. Clin Chem.

[29] Pattison DI, Hawkins CL, Davies MJ (2009). What are the plasma targets of the oxidant hypochlorous acid? A kinetic modeling approach. Chem Res Toxicol.

[30] Taylor JC, Oey L (1982). Ceruloplasmin: plasma inhibitor of the oxidative inactivation of alpha 1-protease inhibitor. Am Rev Respir Dis.

[31] Baskol G, Demir H, Baskol M, Kilic E, Ates F, Karakukcu C, Ustdal M (2006). Investigation of protein oxidation and lipid peroxidation in patients with rheumatoid arthritis. Cell Biochem Funct.

[32] Witko-Sarsat V, Friedlander M, Nguyen-Khoa T, Capeillère-Blandin C, Nguyen AT, Canteloup S, Dayer JM, Jungers P, Drüeke T, Descamps-Latscha B (1998). Advanced oxidation protein products as novel mediators of inflammation and monocyte activation in chronic renal failure. J Immunol.

[33] Liu SX, Hou FF, Guo ZJ, Nagai R, Zhang WR, Liu ZQ, Zhou ZM, Zhou M, Xie D, Wang GB, Zhang X (2006). Advanced oxidation protein products accelerate atherosclerosis through promoting oxidative stress and inflammation. Arterioscler Thromb Vasc Biol.

[34] Descamps-Latscha B, Witko-Sarsat V (2001). Importance of oxidatively modified proteins in chronic renal failure. Kidney Int Suppl.

[35] Cao W, Hou FF, Nie J (2014). AOPPs and the progression of kidney disease. Kidney Int Suppl.

[36] Witko-Sarsat V, Gausson V, Nguyen A-T, Touam M, Drüeke T, Santangelo F, Descamps-Latscha B (2003). AOPP-induced activation of human neutrophil and monocyte oxidative metabolism: a potential target for N-acetylcysteine treatment in dialysis patients. Kidney Int.

[37] Ulfig A, Schulz AV, Müller A, Lupilov N, Leichert LI (2019). N-chlorination mediates protective and immunomodulatory effects of oxidized human plasma proteins. Elife.

[38] Gorudko IV, Grigorieva DV, Shamova EV, Kostevich VA, Sokolov AV, Mikhalchik EV, Cherenkevich SN, Arnhold J, Panasenko OM (2014). Hypohalous acid-modified human serum albumin induces neutrophil NADPH oxidase activation, degranulation, and shape change. Free Radic Biol Med.

[39] Wyatt AR, Kumita JR, Mifsud RW, Gooden CA, Wilson MR, Dobson CM (2014). Hypochlorite-induced structural modifications enhance the chaperone activity of human 2-macroglobulin. Proc Natl Acad Sci.

[40] Dancey JT, Deubelbeiss KA, Harker LA, Finch CA (1976). Neutrophil kinetics in man. J Clin Invest.

[41] Yvan-Charvet L, Ng LG (2019). Granulopoiesis and neutrophil homeostasis: a metabolic, daily balancing act. Trends Immunol.

[42] Hidalgo A, Chilvers ER, Summers C, Koenderman L (2019). The neutrophil life cycle. Trends Immunol.

[43] Tak T, Tesselaar K, Pillay J, Borghans JAM, Koenderman L (2013). What’s your age again? Determination of human neutrophil half-lives revisited. J Leukoc Biol.

[44] Pillay J, den Braber I, Vrisekoop N, Kwast LM, de Boer RJ, Borghans JAM, Tesselaar K, Koenderman L (2010). In vivo labeling with 2H_2_O reveals a human neutrophil lifespan of 5.4 days. Blood.

[45] Cheretakis C, Leung R, Sun CX, Dror Y, Glogauer M (2006). Timing of neutrophil tissue repopulation predicts restoration of innate immune protection in a murine bone marrow transplantation model. Blood.

[46] Geering B, Stoeckle C, Conus S, Simon H-U (2013). Living and dying for inflammation: neutrophils, eosinophils, basophils. Trends Immunol.

[47] Sundqvist M, Wekell P, Osla V, Bylund J, Christenson K, Sävman K, Foell D, Cabral DA, Fasth A, Berg S, Brown KL, Karlsson A (2013). Increased intracellular oxygen radical production in neutrophils during febrile episodes of periodic fever, aphthous stomatitis, pharyngitis, and cervical adenitis syndrome. Arthritis Rheum.

[48] Colotta F, Re F, Polentarutti N, Sozzani S, Mantovani A (1992). Modulation of granulocyte survival and programmed cell death by cytokines and bacterial products. Blood.

[49] Uddin M, Nong G, Ward J, Seumois G, Prince LR, Wilson SJ, Cornelius V, Dent G, Djukanovic R (2010). Prosurvival activity for airway neutrophils in severe asthma. Thorax.

[50] Garlichs CD, Eskafi S, Cicha I, Schmeisser A, Walzog B, Raaz D, Stumpf C, Yilmaz A, Bremer J, Ludwig J, Daniel WG (2004). Delay of neutrophil apoptosis in acute coronary syndromes. J Leukoc Biol.

[51] Bratton DL, Henson PM (2011). Neutrophil clearance: when the party is over, clean-up begins. Trends Immunol.

[52] Martin C, Burdon PCE, Bridger G, Gutierrez-Ramos JC, Williams TJ, Rankin SM (2003). Chemokines acting via CXCR2 and CXCR4 control the release of neutrophils from the bone marrow and their return following senescence. Immunity.

[53] Margraf A, Ley K, Zarbock A (2019). Neutrophil recruitment: from model systems to tissue-specific patterns. Trends Immunol.

[54] Filippi M-D (2019). Neutrophil transendothelial migration: updates and new perspectives. Blood.

[55] Rajaee A, Barnett R, Cheadle WG (2018). Pathogen- and danger-associated molecular patterns and the cytokine response in sepsis. Surg Infect (Larchmt).

[56] Pittman K, Kubes P (2013). Damage-associated molecular patterns control neutrophil recruitment. J Innate Immun.

[57] Huang C, Niethammer P (2018). Tissue damage signaling is a prerequisite for protective neutrophil recruitment to microbial infection in Zebrafish. Immunity.

[58] Mogensen TH (2009). Pathogen recognition and inflammatory signaling in innate immune defenses. Clin Microbiol Rev.

[59] Sundd P, Gutierrez E, Koltsova EK, Kuwano Y, Fukuda S, Pospieszalska MK, Groisman A, Ley K (2012). “Slings” enable neutrophil rolling at high shear. Nature.

[60] Marki A, Buscher K, Mikulski Z, Pries A, Ley K (2018). Rolling neutrophils form tethers and slings under physiologic conditions in vivo. J Leukoc Biol.

[61] Alon R, Fuhlbrigge RC, Finger EB, Springer TA (1996). Interactions through L-selectin between leukocytes and adherent leukocytes nucleate rolling adhesions on selectins and VCAM-1 in shear flow. J Cell Biol.

[62] Charo IF, Yuen C, Goldstein IM (1985). Adherence of human polymorphonuclear leukocytes to endothelial monolayers: effects of temperature, divalent cations, and chemotactic factors on the strength of adherence measured with a new centrifugation assay. Blood.

[63] Langereis JD (2013). Neutrophil integrin affinity regulation in adhesion, migration, and bacterial clearance. Cell Adhesion Migrate.

[64] Vestweber D (2012). Relevance of endothelial junctions in leukocyte extravasation and vascular permeability. Ann N Y Acad Sci.

[65] Yamashiro S, Kamohara H, Wang JM, Yang D, Gong WH, Yoshimura T (2001). Phenotypic and functional change of cytokine-activated neutrophils: inflammatory neutrophils are heterogeneous and enhance adaptive immune responses. J Leukoc Biol.

[66] Lee A, Whyte MK, Haslett C (1993). Inhibition of apoptosis and prolongation of neutrophil functional longevity by inflammatory mediators. J Leukoc Biol.

[67] Takano T, Azuma N, Satoh M, Toda A, Hashida Y, Satoh R, Hohdatsu T (2009). Neutrophil survival factors (TNF-alpha, GM-CSF, and G-CSF) produced by macrophages in cats infected with feline infectious peritonitis virus contribute to the pathogenesis of granulomatous lesions. Arch Virol.

[68] de Haas CJC, Veldkamp KE, Peschel A, Weerkamp F, Van Wamel WJB, Heezius ECJM, Poppelier MJJG, Van Kessel KPM, van Strijp JAG (2004). Chemotaxis inhibitory protein of *Staphylococcus aureus,* a bacterial antiinflammatory agent. J Exp Med.

[69] Bardoel BW, Vos R, Bouman T, Aerts PC, Bestebroer J, Huizinga EG, Brondijk THC, van Strijp JAG, de Haas CJC (2012). Evasion of Toll-like receptor 2 activation by staphylococcal superantigen-like protein 3. J Mol Med.

[70] Laarman AJ, Mijnheer G, Mootz JM, van Rooijen WJM, Ruyken M, Malone CL, Heezius EC, Ward R, Milligan G, van Strijp JAG, de Haas CJC, Horswill AR, van Kessel KPM, Rooijakkers SHM (2012). *Staphylococcus aureus* Staphopain A inhibits CXCR2-dependent neutrophil activation and chemotaxis. EMBO J.

[71] Zinkernagel AS, Timmer AM, Pence MA, Locke JB, Buchanan JT, Turner CE, Mishalian I, Sriskandan S, Hanski E, Nizet V (2008). The IL-8 protease SpyCEP/ScpC of group A Streptococcus promotes resistance to neutrophil killing. Cell Host Microbe.

[72] Döhrmann S, Cole JN, Nizet V (2016). Conquering neutrophils. PLOS Pathog.

[73] Guerra FE, Borgogna TR, Patel DM, Sward EW, Voyich JM (2017). Epic immune battles of history: neutrophils vs. *Staphylococcus aureus*. Front Cell Infect Microbiol.

[74] Teng T-S, Ji A-L, Ji X-Y, Li Y-Z (2017). Neutrophils and immunity: from bactericidal action to being conquered. J Immunol Res.

[75] Abi Abdallah DS, Denkers EY (2012). Neutrophils cast extracellular traps in response to protozoan parasites. Front Immunol.

[76] Barger SR, Gauthier NC, Krendel M (2020). Squeezing in a Meal: myosin Functions in Phagocytosis. Trends Cell Biol.

[77] Thomas DC (2017). The phagocyte respiratory burst: historical perspectives and recent advances. Immunol Lett.

[78] Garin J, Diez R, Kieffer S, Dermine J-F, Duclos S, Gagnon E, Sadoul R, Rondeau C, Desjardins M (2001). The phagosome proteome. J Cell Biol.

[79] Pitt A, Mayorga LS, Stahl PD, Schwartz AL (1992). Alterations in the protein composition of maturing phagosomes. J Clin Invest.

[80] Hackam DJ, Rotstein OD, Zhang W-J, Demaurex N, Woodside M, Tsai O, Grinstein S (1997). Regulation of phagosomal acidification. J Biol Chem.

[81] Schröder BA, Wrocklage C, Hasilik A, Saftig P (2010). The proteome of lysosomes. Proteomics.

[82] Steinberg BE, Huynh KK, Brodovitch A, Jabs S, Stauber T, Jentsch TJ, Grinstein S (2010). A cation counterflux supports lysosomal acidification. J Cell Biol.

[83] Cech P, Lehrer RI (1984). Phagolysosomal pH of human neutrophils. Blood.

[84] Jankowski A, Scott CC, Grinstein S (2002). Determinants of the phagosomal pH in neutrophils. J Biol Chem.

[85] Segal AW (1996). The NADPH oxidase and chronic granulomatous disease. Mol Med Today.

[86] Cox JA, Jeng AY, Sharkey NA, Blumberg PM, Tauber AI (1985). Activation of the human neutrophil nicotinamide adenine dinucleotide phosphate (NADPH)-oxidase by protein kinase C. J Clin Invest.

[87] Winterbourn CC, Kettle AJ (2013). Redox reactions and microbial killing in the neutrophil phagosome. Antioxid Redox Signal.

[88] Quinn MT, Gauss KA (2004). Structure and regulation of the neutrophil respiratory burst oxidase: comparison with nonphagocyte oxidases. J Leukoc Biol.

[89] Bielski BHJ, Cabelli DE, Arudi RL, Ross AB (1985). Reactivity of HO2/O − 2 radicals in aqueous solution. J Phys Chem Ref Data.

[90] Winterbourn CC, Hampton MB, Livesey JH, Kettle AJ (2006). Modeling the reactions of superoxide and myeloperoxidase in the neutrophil phagosome: implications for microbial killing. J Biol Chem.

[91] De Grey A (2002). HO2*: the forgotten radical. DNA Cell Biol.

[92] Buettner GR (1993). The pecking order of free radicals and antioxidants: lipid peroxidation, alpha-tocopherol, and ascorbate. Arch Biochem Biophys.

[93] Gerasimov OV, Lymar SV (1999). The yield of hydroxyl radical from the decomposition of peroxynitrous acid. Inorg Chem.

[94] Crow JP, Spruell C, Chen J, Gunn C, Ischiropoulos H, Tsai M, Smith CD, Radi R, Koppenol WH, Beckman JS (1994). On the pH-dependent yield of hydroxyl radical products from peroxynitrite. Free Radic Biol Med.

[95] Nathan C, Ding A (2010). Snapshot: reactive oxygen intermediates (ROI). Cell.

[96] Halliwell B (2006). Phagocyte-derived reactive species: salvation or suicide?. Trends Biochem Sci.

[97] Fang FC (2004). Antimicrobial reactive oxygen and nitrogen species: concepts and controversies. Nat Rev Microbiol.

[98] Segal BH, Leto TL, Gallin JI, Malech HL, Holland SM (2000). Genetic, biochemical, and clinical features of chronic granulomatous disease. Medicine (Baltimore).

[99] Roos D (2016). Chronic granulomatous disease. Br Med Bull.

[100] Roos D, de Boer M (2014). Molecular diagnosis of chronic granulomatous disease. Clin Exp Immunol.

[101] Aratani Y, Kura F, Watanabe H, Akagawa H, Takano Y, Suzuki K, Dinauer MC, Maeda N, Koyama H (2002). Relative contributions of myeloperoxidase and NADPH-oxidase to the early host defense against pulmonary infections with *Candida albicans* and *Aspergillus fumigatus*. Med Mycol.

[102] Segal AW (2005). How neutrophils kill microbes. Annu Rev Immunol.

[103] Slauch JM (2011). How does the oxidative burst of macrophages kill bacteria? Still an open question. Mol Microbiol.

[104] Stapels DAC, Geisbrecht BV, Rooijakkers SHM (2015). Neutrophil serine proteases in antibacterial defense. Curr Opin Microbiol.

[105] Reeves EP, Lu H, Jacobs HL, Messina CGM, Bolsover S, Gabella G, Potma EO, Warley A, Roes J, Segal AW (2002). Killing activity of neutrophils is mediated through activation of proteases by K + flux. Nature.

[106] Paiva CN, Bozza MT (2014). Are reactive oxygen species always detrimental to pathogens?. Antioxid Redox Signal.

[107] Bryan N, Ahswin H, Smart N, Bayon Y, Wohlert S, Hunt J (2012). Reactive oxygen species (ROS)—a family of fate deciding molecules pivotal in constructive inflammation and wound healing. Eur Cells Mater.

[108] Matsuzawa A, Saegusa K, Noguchi T, Sadamitsu C, Nishitoh H, Nagai S, Koyasu S, Matsumoto K, Takeda K, Ichijo H (2005). ROS-dependent activation of the TRAF6-ASK1-p38 pathway is selectively required for TLR4-mediated innate immunity. Nat Immunol.

[109] Brinkmann V, Reichard U, Goosmann C, Fauler B, Uhlemann Y, Weiss DS, Weinrauch Y, Zychlinsky A (2004). Neutrophil extracellular traps kill bacteria. Science.

[110] Tal MC, Sasai M, Lee HK, Yordy B, Shadel GS, Iwasaki A (2009). Absence of autophagy results in reactive oxygen species-dependent amplification of RLR signaling. Proc Natl Acad Sci USA.

[111] Huang J, Canadien V, Lam GY, Steinberg BE, Dinauer MC, Magalhaes MAO, Glogauer M, Grinstein S, Brumell JH (2009). Activation of antibacterial autophagy by NADPH oxidases. Proc Natl Acad Sci USA.

[112] Huang J, Lam GY, Brumell JH (2011). Autophagy signaling through reactive oxygen species. Antioxid Redox Signal.

[113] Niethammer P, Grabher C, Look AT, Mitchison TJ (2009). A tissue-scale gradient of hydrogen peroxide mediates rapid wound detection in zebrafish. Nature.

[114] Yoo SK, Starnes TW, Deng Q, Huttenlocher A (2011). Lyn is a redox sensor that mediates leukocyte wound attraction in vivo. Nature.

[115] Martinon F (2010). Signaling by ROS drives inflammasome activation. Eur J Immunol.

[116] Cho YS, Challa S, Moquin D, Genga R, Ray TD, Guildford M, Chan FK-M (2009). Phosphorylation-driven assembly of the RIP1-RIP3 complex regulates programmed necrosis and virus-induced inflammation. Cell.

[117] Ashida H, Mimuro H, Ogawa M, Kobayashi T, Sanada T, Kim M, Sasakawa C (2011). Cell death and infection: a double-edged sword for host and pathogen survival. J Cell Biol.

[118] Chan RC-F, Wang M, Li N, Yanagawa Y, Onoé K, Lee JJ, Nel AE (2006). Pro-oxidative diesel exhaust particle chemicals inhibit LPS-induced dendritic cell responses involved in T-helper differentiation. J Allergy Clin Immunol.

[119] Mantegazza AR, Savina A, Vermeulen M, Pérez L, Geffner J, Hermine O, Rosenzweig SD, Faure F, Amigorena S (2008). NADPH oxidase controls phagosomal pH and antigen cross-presentation in human dendritic cells. Blood.

[120] Tang H, Cao W, Kasturi SP, Ravindran R, Nakaya HI, Kundu K, Murthy N, Kepler TB, Malissen B, Pulendran B (2010). The T helper type 2 response to cysteine proteases requires dendritic cell-basophil cooperation via ROS-mediated signaling. Nat Immunol.

[121] Yarosz EL, Chang C-H (2018). The role of reactive oxygen species in regulating T Cell-mediated immunity and disease. Immune Netw.

[122] Tkalcevic J, Novelli M, Phylactides M, Iredale JP, Segal AW, Roes J (2000). Impaired immunity and enhanced resistance to endotoxin in the absence of neutrophil Elastase and Cathepsin G. Immunity.

[123] Belaaouaj A, McCarthy R, Baumann M, Gao Z, Ley TJ, Abraham SN, Shapiro SD (1998). Mice lacking neutrophil elastase reveal impaired host defense against gram negative bacterial sepsis. Nat Med.

[124] Fuchs TA, Abed U, Goosmann C, Hurwitz R, Schulze I, Wahn V, Weinrauch Y, Brinkmann V, Zychlinsky A (2007). Novel cell death program leads to neutrophil extracellular traps. J Cell Biol.

[125] Estúa-Acosta GA, Zamora-Ortiz R, Buentello-Volante B, García-Mejía M, Garfias Y (2019). Neutrophil extracellular traps: current perspectives in the eye. Cells.

[126] Urban CF, Ermert D, Schmid M, Abu-Abed U, Goosmann C, Nacken W, Brinkmann V, Jungblut PR, Zychlinsky A (2009). Neutrophil extracellular traps contain calprotectin, a cytosolic protein complex involved in host defense against Candida albicans. PLoS Pathog.

[127] Brinkmann V, Zychlinsky A (2012). Neutrophil extracellular traps: is immunity the second function of chromatin?. J Cell Biol.

[128] Sollberger G, Tilley DO, Zychlinsky A (2018). Neutrophil extracellular traps: the biology of chromatin externalization. Dev Cell.

[129] Parker H, Albrett AM, Kettle AJ, Winterbourn CC (2012). Myeloperoxidase associated with neutrophil extracellular traps is active and mediates bacterial killing in the presence of hydrogen peroxide. J Leukoc Biol.

[130] Parker H, Winterbourn CC (2012). Reactive oxidants and myeloperoxidase and their involvement in neutrophil extracellular traps. Front Immunol.

[131] Brinkmann V (2018). Neutrophil extracellular traps in the second decade. J Innate Immun.

[132] Xie K, Varatnitskaya M, Maghnouj A, Bader V, Winklhofer KF, Hahn S, Leichert LI (2020). Activation leads to a significant shift in the intracellular redox homeostasis of neutrophil-like cells. Redox Biol.

[133] Yost CC, Cody MJ, Harris ES, Thornton NL, McInturff AM, Martinez ML, Chandler NB, Rodesch CK, Albertine KH, Petti CA, Weyrich AS, Zimmerman GA (2009). Impaired neutrophil extracellular trap (NET) formation: a novel innate immune deficiency of human neonates. Blood.

[134] Parker H, Dragunow M, Hampton MB, Kettle AJ, Winterbourn CC (2012). Requirements for NADPH oxidase and myeloperoxidase in neutrophil extracellular trap formation differ depending on the stimulus. J Leukoc Biol.

[135] Ermert D, Urban CF, Laube B, Goosmann C, Zychlinsky A, Brinkmann V (2009). Mouse neutrophil extracellular traps in microbial infections. J Innate Immun.

[136] Yipp BG, Kubes P (2013). NETosis: how vital is it?. Blood.

[137] Masuda S, Nakazawa D, Shida H, Miyoshi A, Kusunoki Y, Tomaru U, Ishizu A (2016). NETosis markers: quest for specific, objective, and quantitative markers. Clin Chim Acta.

[138] Röhm M, Grimm MJ, D’Auria AC, Almyroudis NG, Segal BH, Urban CF (2014). NADPH oxidase promotes neutrophil extracellular trap formation in pulmonary aspergillosis. Infect Immun.

[139] Bianchi M, Hakkim A, Brinkmann V, Siler U, Seger RA, Zychlinsky A, Reichenbach J (2009). Restoration of NET formation by gene therapy in CGD controls aspergillosis. Blood.

[140] Papayannopoulos V, Metzler KD, Hakkim A, Zychlinsky A (2010). Neutrophil elastase and myeloperoxidase regulate the formation of neutrophil extracellular traps. J Cell Biol.

[141] Neubert E, Bach KM, Busse J, Bogeski I, Schön MP, Kruss S, Erpenbeck L (2019). Blue and long-wave ultraviolet light induce in vitro Neutrophil Extracellular Trap (NET) Formation. Front Immunol.

[142] Martinod K, Witsch T, Farley K, Gallant M, Remold-O’Donnell E, Wagner DD (2016). Neutrophil elastase-deficient mice form neutrophil extracellular traps in an experimental model of deep vein thrombosis. J Thromb Haemost.

[143] Storisteanu DML, Pocock JM, Cowburn AS, Juss JK, Nadesalingam A, Nizet V, Chilvers ER (2017). Evasion of neutrophil extracellular traps by respiratory pathogens. Am J Respir Cell Mol Biol.

[144] Eby JC, Gray MC, Hewlett EL (2014). Cyclic AMP-mediated suppression of neutrophil extracellular trap formation and apoptosis by the Bordetella pertussis adenylate cyclase toxin. Infect Immun.

[145] Carlin AF, Uchiyama S, Chang Y-C, Lewis AL, Nizet V, Varki A (2009). Molecular mimicry of host sialylated glycans allows a bacterial pathogen to engage neutrophil Siglec-9 and dampen the innate immune response. Blood.

[146] Buchanan JT, Simpson AJ, Aziz RK, Liu GY, Kristian SA, Kotb M, Feramisco J, Nizet V (2006). DNase expression allows the pathogen group A Streptococcus to escape killing in neutrophil extracellular traps. Curr Biol.

[147] Beiter K, Wartha F, Albiger B, Normark S, Zychlinsky A, Henriques-Normark B (2006). An endonuclease allows Streptococcus pneumoniae to escape from neutrophil extracellular traps. Curr Biol.

[148] Wartha F, Beiter K, Albiger B, Fernebro J, Zychlinsky A, Normark S, Henriques-Normark B (2007). Capsule and D-alanylated lipoteichoic acids protect Streptococcus pneumoniae against neutrophil extracellular traps. Cell Microbiol.

[149] Schultz J, Kaminker K (1962). Myeloperoxidase of the leucocyte of normal human blood. I. Content and localization. Arch Biochem Biophys.

[150] Klebanoff SJ, Kettle AJ, Rosen H, Winterbourn CC, Nauseef WM (2013). Myeloperoxidase: a front-line defender against phagocytosed microorganisms. J Leukoc Biol.

[151] Hampton MB, Kettle AJ, Winterbourn CC (1998). Inside the neutrophil phagosome: oxidants, myeloperoxidase, and bacterial killing. Blood.

[152] Kettle AJ, Winterbourn CC (1994) Assays for the chlorination activity of myeloperoxidase. In: Methods in enzymology. pp 502–512 10.1016/s0076-6879(94)33056-5 8015486

[153] Weiss SJ (1989). Tissue destruction by neutrophils. N Engl J Med.

[154] Dunford HB (1987). Free radicals in iron-containing systems. Free Radic Biol Med.

[155] Marquez LA, Dunford HB (1995). Kinetics of oxidation of tyrosine and dityrosine by myeloperoxidase compounds I and II. J Biol Chem.

[156] Heinecke JW, Li W, Daehnke HL, Goldstein JA (1993). Dityrosine, a specific marker of oxidation, is synthesized by the myeloperoxidase-hydrogen peroxide system of human neutrophils and macrophages. J Biol Chem.

[157] Afshinnia F, Zeng L, Byun J, Gadegbeku CA, Magnone MC, Whatling C, Valastro B, Kretzler M, Pennathur S, Gcxvroup MKTCCI (2017). Myeloperoxidase levels and its product 3-chlorotyrosine predict chronic kidney disease severity and associated coronary artery disease. Am J Nephrol.

[158] O’Donnell C, Newbold P, White P, Thong B, Stone H, Stockley RA (2010). 3-Chlorotyrosine in sputum of COPD patients: relationship with airway inflammation. COPD.

[159] Cheng G, Salerno JC, Cao Z, Pagano PJ, Lambeth JD (2008). Identification and characterization of VPO1, a new animal heme-containing peroxidase. Free Radic Biol Med.

[160] Li H, Cao Z, Moore DR, Jackson PL, Barnes S, Lambeth JD, Thannickal VJ, Cheng G (2012). Microbicidal activity of vascular peroxidase 1 in human plasma via generation of hypochlorous acid. Infect Immun.

[161] McCall AS, Cummings CF, Bhave G, Vanacore R, Page-McCaw A, Hudson BG (2014). Bromine is an essential trace element for assembly of collagen IV scaffolds in tissue development and architecture. Cell.

[162] Colon S, Page-McCaw P, Bhave G (2017). Role of hypohalous acids in basement membrane homeostasis. Antioxid Redox Signal.

[163] Paumann-Page M, Katz RS, Bellei M, Schwartz I, Edenhofer E, Sevcnikar B, Soudi M, Hofbauer S, Battistuzzi G, Furtmüller PG, Obinger C (2017). Pre-steady-state kinetics reveal the substrate specificity and mechanism of halide oxidation of truncated human peroxidasin 1. J Biol Chem.

[164] Tavora FR, Ripple M, Li L, Burke AP (2009). Monocytes and neutrophils expressing myeloperoxidase occur in fibrous caps and thrombi in unstable coronary plaques. BMC Cardiovasc Disord.

[165] Daugherty A, Dunn JL, Rateri DL, Heinecke JW (1994). Myeloperoxidase, a catalyst for lipoprotein oxidation, is expressed in human atherosclerotic lesions. J Clin Invest.

[166] Chase MJ, Klebanoff SJ (1992). Viricidal effect of stimulated human mononuclear phagocytes on human immunodeficiency virus type 1. Proc Natl Acad Sci.

[167] Karhumäki E, Helin H (1987). Regulation of oxidative metabolism by interferon-gamma during human monocyte to macrophage differentiation. Med Biol.

[168] Nakagawara A, Nathan CF, Cohn ZA (1981). Hydrogen peroxide metabolism in human monocytes during differentiation in vitro. J Clin Invest.

[169] Kumar AP, Piedrafita FJ, Reynolds WF (2004). Peroxisome proliferator-activated receptor gamma ligands regulate myeloperoxidase expression in macrophages by an estrogen-dependent mechanism involving the -463GA promoter polymorphism. J Biol Chem.

[170] Klebanoff SJ (2005). Myeloperoxidase: friend and foe. J Leukoc Biol.

[171] Brown KE, Brunt EM, Heinecke JW (2001). Immunohistochemical detection of myeloperoxidase and its oxidation products in Kupffer cells of human liver. Am J Pathol.

[172] Nagra RM, Becher B, Tourtellotte WW, Antel JP, Gold D, Paladino T, Smith RA, Nelson JR, Reynolds WF (1997). Immunohistochemical and genetic evidence of myeloperoxidase involvement in multiple sclerosis. J Neuroimmunol.

[173] Sugiyama S, Okada Y, Sukhova GK, Virmani R, Heinecke JW, Libby P (2001). Macrophage myeloperoxidase regulation by granulocyte macrophage colony-stimulating factor in human atherosclerosis and implications in acute coronary syndromes. Am J Pathol.

[174] Shepherd VL, Hoidal JR (1990). Clearance of neutrophil-derived myeloperoxidase by the macrophage mannose receptor. Am J Respir Cell Mol Biol.

[175] Breckwoldt MO, Chen JW, Stangenberg L, Aikawa E, Rodriguez E, Qiu S, Moskowitz MA, Weissleder R (2008). Tracking the inflammatory response in stroke in vivo by sensing the enzyme myeloperoxidase. Proc Natl Acad Sci.

[176] Okada SS, de Oliveira EM, de Araújo TH, Rodrigues MR, Albuquerque RC, Mortara RA, Taniwaki NN, Nakaya HI, Campa A, Moreno ACR (2016). Myeloperoxidase in human peripheral blood lymphocytes: production and subcellular localization. Cell Immunol.

[177] de Araújo TH, Okada SS, Ghosn EEB, Taniwaki NN, Rodrigues MR, de Almeida SR, Mortara RA, Russo M, Campa A, Albuquerque RC (2013). Intracellular localization of myeloperoxidase in murine peritoneal B-lymphocytes and macrophages. Cell Immunol.

[178] Klebanoff SJ, Hamon CB (1972). Role of myeloperoxidase-mediated antimicrobial systems in intact leukocytes. J Reticuloendothel Soc.

[179] Degrossoli A, Müller A, Xie K, Schneider JF, Bader V, Winklhofer KF, Meyer AJ, Leichert LI (2018). Neutrophil-generated HOCl leads to non-specific thiol oxidation in phagocytized bacteria. Elife.

[180] Lehrer RI, Hanifin J, Cline MJ (1969). Defective bactericidal activity in myeloperoxidase-deficient human neutrophils. Nature.

[181] Metzler KD, Fuchs TA, Nauseef WM, Reumaux D, Roesler J, Schulze I, Wahn V, Papayannopoulos V, Zychlinsky A (2011). Myeloperoxidase is required for neutrophil extracellular trap formation: implications for innate immunity. Blood.

[182] Ludviksson BR, Thorarensen O, Gudnason T, Halldorsson S (1993). Candida albicans meningitis in a child with myeloperoxidase deficiency. Pediatr Infect Dis J.

[183] Nauseef WM (1988). Myeloperoxidase deficiency. Hematol Oncol Clin North Am.

[184] Okuda T, Yasuoka T, Oka N (1991). Myeloperoxidase deficiency as a predisposing factor for deep mucocutaneous candidiasis: a case report. J Oral Maxillofac Surg.

[185] Diamond RD, Clark RA (1982). Damage to *Aspergillus fumigatus* and *Rhizopus oryzae* hyphae by oxidative and nonoxidative microbicidal products of human neutrophils in vitro. Infect Immun.

[186] Cech P, Stalder H, Widmann JJ, Rohner A, Miescher PA (1979). Leukocyte myeloperoxidase deficiency and diabetes mellitus associated with *Candida albicans* liver abscess. Am J Med.

[187] Koziol-Montewka M, Magrys A, Paluch-Oles J, Bogut A, Buczynski K, Jablonka S (2006). MPO and cytokines in the serum of cancer patients in the context of Candida colonization and infection. Immunol Invest.

[188] Rosen H, Klebanoff SJ (1976). Chemiluminescence and superoxide production by myeloperoxidase-deficient leukocytes. J Clin Invest.

[189] Klebanoff SJ, Pincus SH (1971). Hydrogen peroxide utilization in myeloperoxidase-deficient leukocytes: a possible microbicidal control mechanism. J Clin Invest.

[190] Nauseef WM, Metcalf JA, Root RK (1983). Role of myeloperoxidase in the respiratory burst of human neutrophils. Blood.

[191] Hampton MB, Kettle AJ, Winterbourn CC (1996). Involvement of superoxide and myeloperoxidase in oxygen-dependent killing of Staphylococcus aureus by neutrophils. Infect Immun.

[192] Zeng J, Fenna RE (1992). X-ray crystal structure of canine myeloperoxidase at 3 A resolution. J Mol Biol.

[193] Olsen RL, Little C (1984). Studies on the subunits of human myeloperoxidase. Biochem J.

[194] Andersen MR, Atkin CL, Eyre HJ (1982). Intact form of myeloperoxidase from normal human neutrophils. Arch Biochem Biophys.

[195] Grishkovskaya I, Paumann-Page M, Tscheliessnig R, Stampler J, Hofbauer S, Soudi M, Sevcnikar B, Oostenbrink C, Furtmüller PG, Djinović-Carugo K, Nauseef WM, Obinger C (2017). Structure of human promyeloperoxidase (proMPO) and the role of the propeptide in processing and maturation. J Biol Chem.

[196] Fiedler TJ, Davey CA, Fenna RE (2000). X-ray crystal structure and characterization of halide-binding sites of human myeloperoxidase at 1.8 A resolution. J Biol Chem.

[197] Battistuzzi G, Stampler J, Bellei M, Vlasits J, Soudi M, Furtmüller PG, Obinger C (2011). Influence of the covalent heme-protein bonds on the redox thermodynamics of human myeloperoxidase. Biochemistry.

[198] Carpena X, Vidossich P, Schroettner K, Calisto BM, Banerjee S, Stampler J, Soudi M, Furtmüller PG, Rovira C, Fita I, Obinger C (2009). Essential role of proximal histidine-asparagine interaction in mammalian peroxidases. J Biol Chem.

[199] Fenna R, Zeng J, Davey C (1995). Structure of the green heme in myeloperoxidase. Arch Biochem Biophys.

[200] Kooter IM, Moguilevsky N, Bollen A, van der Veen LA, Otto C, Dekker HL, Wever R (1999). The sulfonium ion linkage in myeloperoxidase. J Biol Chem.

[201] Kooter IM, Moguilevsky N, Bollen A, Sijtsema NM, Otto C, Wever R (1997). Site-directed mutagenesis of Met243, a residue of myeloperoxidase involved in binding of the prosthetic group. JBIC, J Biol Inorg Chem.

[202] Floris R, Moguilevsky N, Puppels G, Jacquet A, Renirie R, Bollen A, Wever R (1995). Heme-protein interaction in myeloperoxidase: modification of spectroscopic properties and catalytic activity by single residue mutation. J Am Chem Soc.

[203] Davies MJ, Hawkins CL, Pattison DI, Rees MD (2008). Mammalian heme peroxidases: from molecular mechanisms to health implications. Antioxid Redox Signal.

[204] Furtmüller PG, Zederbauer M, Jantschko W, Helm J, Bogner M, Jakopitsch C, Obinger C (2006). Active site structure and catalytic mechanisms of human peroxidases. Arch Biochem Biophys.

[205] Davies MJ (2011). Myeloperoxidase-derived oxidation: mechanisms of biological damage and its prevention. J Clin Biochem Nutr.

[206] Sbarra AJ (1979). The neutrophil: function and clinical disorders. Trends Biochem Sci.

[207] Oka S, Sibazaki Y, Tahara S (1981). Direct potentiometric determination of chloride ion in whole blood. Anal Chem.

[208] Holzbecher J, Ryan DE (1980). The rapid determination of total bromine and iodine in biological fluids by neutron activation. Clin Biochem.

[209] Wood JL (1975). Chemistry and biochemistry of thiocyanic acid and its derivatives.

[210] Foote CS, Goyne TE, Lehrer RI (1983). Assessment of chlorination by human neutrophils. Nature.

[211] Segal AW, Garcia RC, Harper AM, Banga JP (1983). Iodination by stimulated human neutrophils. Studies on its stoichiometry, subcellular localization and relevance to microbial killing. Biochem J.

[212] Morgan PE, Pattison DI, Talib J, Summers FA, Harmer JA, Celermajer DS, Hawkins CL, Davies MJ (2011). High plasma thiocyanate levels in smokers are a key determinant of thiol oxidation induced by myeloperoxidase. Free Radic Biol Med.

[213] Ashby MT, Carlson AC, Scott MJ (2004). Redox buffering of hypochlorous acid by thiocyanate in physiologic fluids. J Am Chem Soc.

[214] Nagy P, Beal JL, Ashby MT (2006). Thiocyanate is an efficient endogenous scavenger of the phagocytic killing agent hypobromous acid. Chem Res Toxicol.

[215] Chapman ALP, Hampton MB, Senthilmohan R, Winterbourn CC, Kettle AJ (2002). Chlorination of bacterial and neutrophil proteins during phagocytosis and killing of Staphylococcus aureus. J Biol Chem.

[216] Rosen H, Crowley JR, Heinecke JW (2002). Human neutrophils use the myeloperoxidase-hydrogen peroxide-chloride system to chlorinate but not nitrate bacterial proteins during phagocytosis. J Biol Chem.

[217] Winterbourn CC, Kettle AJ, Hampton MB (2016). Reactive oxygen species and neutrophil function. Annu Rev Biochem.

[218] Rosen H, Klebanoff SJ (1979). Bactericidal activity of a superoxide anion-generating system. A model for the polymorphonuclear leukocyte. J Exp Med.

[219] Klebanoff SJ (1980). Oxygen metabolism and the toxic properties of phagocytes. Ann Intern Med.

[220] Giorgio M, Trinei M, Migliaccio E, Pelicci PG (2007). Hydrogen peroxide: a metabolic by-product or a common mediator of ageing signals?. Nat Rev Mol Cell Biol.

[221] Schürmann N, Forrer P, Casse O, Li J, Felmy B, Burgener A-V, Ehrenfeuchter N, Hardt W-D, Recher M, Hess C, Tschan-Plessl A, Khanna N, Bumann D (2017). Myeloperoxidase targets oxidative host attacks to Salmonella and prevents collateral tissue damage. Nat Microbiol.

[222] Bienert GP, Schjoerring JK, Jahn TP (2006). Membrane transport of hydrogen peroxide. Biochim Biophys Acta.

[223] Grisham MB, Jefferson MM, Melton DF, Thomas EL (1984). Chlorination of endogenous amines by isolated neutrophils. Ammonia-dependent bactericidal, cytotoxic, and cytolytic activities of the chloramines. J Biol Chem.

[224] Carr AC, van den Berg JJ, Winterbourn CC (1996). Chlorination of cholesterol in cell membranes by hypochlorous acid. Arch Biochem Biophys.

[225] Hawkins CL (2019). Hypochlorous acid-mediated modification of proteins and its consequences. Essays Biochem.

[226] Davies MJ (2005). The oxidative environment and protein damage. Biochim Biophys Acta.

[227] Davies MJ (2016). Protein oxidation and peroxidation. Biochem J.

[228] Pattison DI, Davies MJ (2001). Absolute rate constants for the reaction of hypochlorous acid with protein side chains and peptide bonds. Chem Res Toxicol.

[229] Storkey C, Davies MJ, Pattison DI (2014). Reevaluation of the rate constants for the reaction of hypochlorous acid (HOCl) with cysteine, methionine, and peptide derivatives using a new competition kinetic approach. Free Radic Biol Med.

[230] Pattison DI, Davies MJ (2004). Kinetic analysis of the reactions of hypobromous acid with protein components: implications for cellular damage and use of 3-bromotyrosine as a marker of oxidative stress. Biochemistry.

[231] Balsera M, Buchanan BB (2019). Evolution of the thioredoxin system as a step enabling adaptation to oxidative stress. Free Radic Biol Med.

[232] Xiao Z, La Fontaine S, Bush AI, Wedd AG (2019). Molecular mechanisms of glutaredoxin enzymes: versatile hubs for thiol-disulfide exchange between protein thiols and glutathione. J Mol Biol.

[233] Biteau B, Labarre J, Toledano MB (2003). ATP-dependent reduction of cysteine–sulphinic acid by S. cerevisiae sulphiredoxin. Nature.

[234] Boileau C, Eme L, Brochier-Armanet C, Janicki A, Zhang C-C, Latifi A (2011). A eukaryotic-like sulfiredoxin involved in oxidative stress responses and in the reduction of the sulfinic form of 2-Cys peroxiredoxin in the cyanobacterium Anabaena PCC 7120. New Phytol.

[235] Skaff O, Pattison DI, Davies MJ (2009). Hypothiocyanous acid reactivity with low-molecular-mass and protein thiols: absolute rate constants and assessment of biological relevance. Biochem J.

[236] Nagy P, Jameson GNL, Winterbourn CC (2009). Kinetics and mechanisms of the reaction of hypothiocyanous acid with 5-thio-2-nitrobenzoic acid and reduced glutathione. Chem Res Toxicol.

[237] Thomas EL, Aune TM (1978). Lactoperoxidase, peroxide, thiocyanate antimicrobial system: correlation of sulfhydryl oxidation with antimicrobial action. Infect Immun.

[238] Skaff O, Pattison DI, Morgan PE, Bachana R, Jain VK, Priyadarsini KI, Davies MJ (2012). Selenium-containing amino acids are targets for myeloperoxidase-derived hypothiocyanous acid: determination of absolute rate constants and implications for biological damage. Biochem J.

[239] Barrett TJ, Hawkins CL (2012). Hypothiocyanous acid: benign or deadly?. Chem Res Toxicol.

[240] Hawkins CL (2009). The role of hypothiocyanous acid (HOSCN) in biological systems. Free Radic Res.

[241] Love DT, Barrett TJ, White MY, Cordwell SJ, Davies MJ, Hawkins CL (2016). Cellular targets of the myeloperoxidase-derived oxidant hypothiocyanous acid (HOSCN) and its role in the inhibition of glycolysis in macrophages. Free Radic Biol Med.

[242] Rosen H, Klebanoff SJ, Wang Y, Brot N, Heinecke JW, Fu X (2009). Methionine oxidation contributes to bacterial killing by the myeloperoxidase system of neutrophils. Proc Natl Acad Sci USA.

[243] Hawkins CL, Pattison DI, Davies MJ (2003). Hypochlorite-induced oxidation of amino acids, peptides and proteins. Amino Acids.

[244] Winterbourn CC, Kettle AJ (2000). Biomarkers of myeloperoxidase-derived hypochlorous acid. Free Radic Biol Med.

[245] Vissers MCM, Winterbourn CC (1991). Oxidative damage to fibronectin. Arch Biochem Biophys.

[246] Hawkins CL, Davies MJ (1998). Hypochlorite-induced damage to proteins: formation of nitrogen-centred radicals from lysine residues and their role in protein fragmentation. Biochem J.

[247] Hazell LJ, van den Berg JJM, Stocker R (1994). Oxidation of low-density lipoprotein by hypochlorite causes aggregation that is mediated by modification of lysine residues rather than lipid oxidation. Biochem J.

[248] Sips HJ, Hamers MN (1981). Mechanism of the bactericidal action of myeloperoxidase: increased permeability of the Escherichia coli cell envelope. Infect Immun.

[249] Venkobachar C, Iyengar L, Prabhakara Rao AVS (1977). Mechanism of disinfection: effect of chlorine on cell membrane functions. Water Res.

[250] Thomas EL (1979). Myeloperoxidase-hydrogen peroxide- chloride antimicrobial system: effect of exogenous amines on antibacterial action against Escherichia coli. Infect Immun.

[251] Bernofsky C (1991). Nucleotide chloramines and neutrophil-mediated cytotoxicity. FASEB J.

[252] Barrette WC, Albrich JM, Hurst JK (1987). Hypochlorous acid-promoted loss of metabolic energy in *Escherichia coli*. Infect Immun.

[253] Albrich JM, Gilbaugh JH, Callahan KB, Hurst JK (1986). Effects of the putative neutrophil-generated toxin, hypochlorous acid, on membrane permeability and transport systems of *Escherichia coli*. J Clin Invest.

[254] Hannum DM, Barrette WC, Hurst JK (1995). Subunit sites of oxidative inactivation of *Escherichia coli* F1-ATPase by HOCl. Biochem Biophys Res Commun.

[255] Khor HK, Fisher MT, Schöneich C (2004). Potential role of methionine sulfoxide in the inactivation of the chaperone GroEL by hypochlorous acid (HOCl) and peroxynitrite (ONOO-). J Biol Chem.

[256] Winter J, Linke K, Jatzek A, Jakob U (2005). Severe oxidative stress causes inactivation of DnaK and activation of the redox-regulated chaperone Hsp33. Mol Cell.

[257] Rosen H, Michel BR, VanDevanter DR, Hughes JP (1998). Differential effects of myeloperoxidase-derived oxidants on *Escherichia coli* DNA replication. Infect Immun.

[258] Imlay JA (2008). Cellular defenses against superoxide and hydrogen peroxide. Annu Rev Biochem.

[259] Ezraty B, Gennaris A, Barras F, Collet J-F (2017). Oxidative stress, protein damage and repair in bacteria. Nat Rev Microbiol.

[260] Gray MJ, Wholey W-Y, Jakob U (2013). Bacterial responses to reactive chlorine species. Annu Rev Microbiol.

[261] Ritz D, Beckwith J (2001). Roles of thiol-redox pathways in bacteria. Annu Rev Microbiol.

[262] Roos G, Messens J (2011). Protein sulfenic acid formation: from cellular damage to redox regulation. Free Radic Biol Med.

[263] Chesney JA, Eaton JW, Mahoney JR (1996). Bacterial glutathione: a sacrificial defense against chlorine compounds. J Bacteriol.

[264] Masip L, Veeravalli K, Georgiou G (2006). The many faces of glutathione in bacteria. Antioxid Redox Signal.

[265] Ceragioli M, Mols M, Moezelaar R, Ghelardi E, Senesi S, Abee T (2010). Comparative transcriptomic and phenotypic analysis of the responses of *Bacillus cereus* to various disinfectant treatments. Appl Environ Microbiol.

[266] Chi BK, Gronau K, Mäder U, Hessling B, Becher D, Antelmann H (2011). S-Bacillithiolation protects against hypochlorite stress in *Bacillus subtilis* as revealed by transcriptomics and redox proteomics. Mol Cell Proteomics.

[267] Small DA, Chang W, Toghrol F, Bentley WE (2007). Toxicogenomic analysis of sodium hypochlorite antimicrobial mechanisms in *Pseudomonas aeruginosa*. Appl Microbiol Biotechnol.

[268] Wang S, Deng K, Zaremba S, Deng X, Lin C, Wang Q, Lou Tortorello M, Zhang W (2009). Transcriptomic response of *Escherichia coli* O157:h7 to oxidative stress. Appl Environ Microbiol.

[269] Weissbach H, Etienne F, Hoshi T, Heinemann SH, Lowther WT, Matthews B, St John G, Nathan C, Brot N (2002). Peptide methionine sulfoxide reductase: structure, mechanism of action, and biological function. Arch Biochem Biophys.

[270] Gebendorfer KM, Drazic A, Le Y, Gundlach J, Bepperling A, Kastenmüller A, Ganzinger KA, Braun N, Franzmann TM, Winter J (2012). Identification of a hypochlorite-specific transcription factor from *Escherichia coli*. J Biol Chem.

[271] Drazic A, Miura H, Peschek J, Le Y, Bach NC, Kriehuber T, Winter J (2013). Methionine oxidation activates a transcription factor in response to oxidative stress. Proc Natl Acad Sci.

[272] Gray MJ, Wholey W-Y, Parker BW, Kim M, Jakob U (2013). NemR is a bleach-sensing transcription factor. J Biol Chem.

[273] Lu S, Killoran PB, Fang FC, Riley LW (2002). The global regulator ArcA controls resistance to reactive nitrogen and oxygen intermediates in *Salmonella enterica* Serovar Enteritidis. Infect Immun.

[274] Morales EH, Calderón IL, Collao B, Gil F, Porwollik S, McClelland M, Saavedra CP (2012). Hypochlorous acid and hydrogen peroxide-induced negative regulation of Salmonella enterica serovar Typhimurium ompW by the response regulator ArcA. BMC Microbiol.

[275] Wong SMS, Alugupalli KR, Ram S, Akerley BJ (2007). The ArcA regulon and oxidative stress resistance in Haemophilus influenzae. Mol Microbiol.

[276] Pardo-Esté C, Hidalgo AA, Aguirre C, Briones AC, Cabezas CE, Castro-Severyn J, Fuentes JA, Opazo CM, Riedel CA, Otero C, Pacheco R, Valvano MA, Saavedra CP (2018). The ArcAB two-component regulatory system promotes resistance to reactive oxygen species and systemic infection by Salmonella Typhimurium. PLoS ONE.

[277] Guisbert E, Herman C, Lu CZ, Gross CA (2004). A chaperone network controls the heat shock response in *E. coli*. Genes Dev.

[278] Arsène F, Tomoyasu T, Bukau B (2000). The heat shock response of *Escherichia coli*. Int J Food Microbiol.

[279] Kumar CMS, Mande SC, Mahajan G (2015). Multiple chaperonins in bacteria–novel functions and non-canonical behaviors. Cell Stress Chaperones.

[280] Georgopoulos C (2006). Toothpicks, serendipity and the emergence of the *Escherichia coli* DnaK (Hsp70) and GroEL (Hsp60) chaperone machines. Genetics.

[281] Houry WA (2001). Chaperone-assisted protein folding in the cell cytoplasm. Curr Protein Pept Sci.

[282] Dahl J-U, Gray MJ, Jakob U (2015). Protein quality control under oxidative stress conditions. J Mol Biol.

[283] Gray MJ, Wholey W-Y, Wagner NO, Cremers CM, Mueller-Schickert A, Hock NT, Krieger AG, Smith EM, Bender RA, Bardwell JCA, Jakob U (2014). Polyphosphate is a primordial chaperone. Mol Cell.

[284] Voth W, Jakob U (2017). Stress-activated chaperones: a first line of defense. Trends Biochem Sci.

[285] Jakob U, Muse W, Eser M, Bardwell JC (1999). Chaperone activity with a redox switch. Cell.

[286] Ilbert M, Horst J, Ahrens S, Winter J, Graf PCF, Lilie H, Jakob U (2007). The redox-switch domain of Hsp33 functions as dual stress sensor. Nat Struct Mol Biol.

[287] Jakob U, Eser M, Bardwell JCA (2000). Redox Switch of Hsp33 Has a Novel Zinc-binding Motif. J Biol Chem.

[288] Voth W, Schick M, Gates S, Li S, Vilardi F, Gostimskaya I, Southworth DR, Schwappach B, Jakob U (2014). The protein targeting factor Get3 functions as ATP-independent chaperone under oxidative stress conditions. Mol Cell.

[289] Powis K, Schrul B, Tienson H, Gostimskaya I, Breker M, High S, Schuldiner M, Jakob U, Schwappach B (2013). Get3 is a holdase chaperone and moves to deposition sites for aggregated proteins when membrane targeting is blocked. J Cell Sci.

[290] Lambrecht JA, Flynn JM, Downs DM (2012). Conserved YjgF Protein family deaminates reactive enamine/imine intermediates of pyridoxal 5′-phosphate (PLP)-dependent enzyme reactions. J Biol Chem.

[291] Johnson RJ, Guggenheim SJ, Klebanoff SJ, Ochi RF, Wass A, Baker P, Schulze M, Couser WG (1988). Morphologic correlates of glomerular oxidant injury induced by the myeloperoxidase-hydrogen peroxide-halide system of the neutrophil. Lab Invest.

[292] Johnson RJ, Couser WG, Chi EY, Adler S, Klebanoff SJ (1987). New mechanism for glomerular injury. Myeloperoxidase-hydrogen peroxide-halide system. J Clin Invest.

[293] Hammerschmidt S, Wahn H (1997). Comparable effects of HOCl and of FMLP-stimulated PMN on the circulation in an isolated lung model. Am J Respir Crit Care Med.

[294] Malech HL, Gallin JI (1987). Current concepts: immunology. Neutrophils in human diseases. N Engl J Med.

[295] Hansson M, Olsson I, Nauseef WM (2006). Biosynthesis, processing, and sorting of human myeloperoxidase. Arch Biochem Biophys.

[296] Wang J-G, Mahmud SA, Nguyen J, Slungaard A (2006). Thiocyanate-dependent induction of endothelial cell adhesion molecule expression by phagocyte peroxidases: a novel HOSCN-specific oxidant mechanism to amplify inflammation. J Immunol.

[297] Wang J, Slungaard A (2006). Role of eosinophil peroxidase in host defense and disease pathology. Arch Biochem Biophys.

[298] Spickett CM, Jerlich A, Panasenko OM, Arnhold J, Pitt AR, Stelmaszyńska T, Schaur RJ (2000). The reactions of hypochlorous acid, the reactive oxygen species produced by myeloperoxidase, with lipids. Acta Biochim Pol.

[299] Barrett TJ, Pattison DI, Leonard SE, Carroll KS, Davies MJ, Hawkins CL (2012). Inactivation of thiol-dependent enzymes by hypothiocyanous acid: role of sulfenyl thiocyanate and sulfenic acid intermediates. Free Radic Biol Med.

[300] Lane AE, Tan JTM, Hawkins CL, Heather AK, Davies MJ (2010). The myeloperoxidase-derived oxidant HOSCN inhibits protein tyrosine phosphatases and modulates cell signalling via the mitogen-activated protein kinase (MAPK) pathway in macrophages. Biochem J.

[301] Lloyd MM, Grima MA, Rayner BS, Hadfield KA, Davies MJ, Hawkins CL (2013). Comparative reactivity of the myeloperoxidase-derived oxidants hypochlorous acid and hypothiocyanous acid with human coronary artery endothelial cells. Free Radic Biol Med.

[302] Lloyd MM, van Reyk DM, Davies MJ, Hawkins CL (2008). Hypothiocyanous acid is a more potent inducer of apoptosis and protein thiol depletion in murine macrophage cells than hypochlorous acid or hypobromous acid. Biochem J.

[303] Xu Y, Szép S, Lu Z (2009). The antioxidant role of thiocyanate in the pathogenesis of cystic fibrosis and other inflammation-related diseases. Proc Natl Acad Sci USA.

[304] Gould NS, Gauthier S, Kariya CT, Min E, Huang J, Brian DJ (2010). Hypertonic saline increases lung epithelial lining fluid glutathione and thiocyanate: two protective CFTR-dependent thiols against oxidative injury. Respir Res.

[305] Hänström L, Johansson A, Carlsson J (1983). Lactoperoxidase and thiocyanate protect cultured mammalian cells against hydrogen peroxide toxicity. Med Biol.

[306] Tenovuo J, Larjava H (1984). The protective effect of peroxidase and thiocyanate against hydrogen peroxide toxicity assessed by the uptake of [3H]-thymidine by human gingival fibroblasts cultured in vitro. Arch Oral Biol.

[307] Talib J, Pattison DI, Harmer JA, Celermajer DS, Davies MJ (2012). High plasma thiocyanate levels modulate protein damage induced by myeloperoxidase and perturb measurement of 3-chlorotyrosine. Free Radic Biol Med.

[308] Nedoboy PE, Morgan PE, Mocatta TJ, Richards AM, Winterbourn CC, Davies MJ (2014). High plasma thiocyanate levels are associated with enhanced myeloperoxidase-induced thiol oxidation and long-term survival in subjects following a first myocardial infarction. Free Radic Res.

[309] Brennan M-L, Penn MS, Van Lente F, Nambi V, Shishehbor MH, Aviles RJ, Goormastic M, Pepoy ML, McErlean ES, Topol EJ, Nissen SE, Hazen SL (2003). Prognostic value of myeloperoxidase in patients with chest pain. N Engl J Med.

[310] Mocatta TJ, Pilbrow AP, Cameron VA, Senthilmohan R, Frampton CM, Richards AM, Winterbourn CC (2007). Plasma concentrations of myeloperoxidase predict mortality after myocardial infarction. J Am Coll Cardiol.

[311] Baldus S, Heeschen C, Meinertz T, Zeiher AM, Eiserich JP, Münzel T, Simoons ML, Hamm CW, Investigators C (2003). Myeloperoxidase serum levels predict risk in patients with acute coronary syndromes. Circulation.

[312] Haegens A, Vernooy JHJ, Heeringa P, Mossman BT, Wouters EFM (2008). Myeloperoxidase modulates lung epithelial responses to pro-inflammatory agents. Eur Respir J.

[313] Klinke A, Nussbaum C, Kubala L, Friedrichs K, Rudolph TK, Rudolph V, Paust H-J, Schröder C, Benten D, Lau D, Szocs K, Furtmüller PG, Heeringa P, Sydow K, Duchstein H-J, Ehmke H, Schumacher U, Meinertz T, Sperandio M, Baldus S (2011). Myeloperoxidase attracts neutrophils by physical forces. Blood.

[314] Lau D, Mollnau H, Eiserich JP, Freeman BA, Daiber A, Gehling UM, Brümmer J, Rudolph V, Münzel T, Heitzer T, Meinertz T, Baldus S (2005). Myeloperoxidase mediates neutrophil activation by association with CD11b/CD18 integrins. Proc Natl Acad Sci U S A.

[315] Summers FA, Morgan PE, Davies MJ, Hawkins CL (2008). Identification of plasma proteins that are susceptible to thiol oxidation by hypochlorous acid and N-chloramines. Chem Res Toxicol.

[316] Arnhold J, Hammerschmidt S, Arnold K (1991). Role of functional groups of human plasma and luminol in scavenging of NaOCl and neutrophil-derived hypochlorous acid. Biochim Biophys Acta Mol Basis Dis.

[317] Hu ML, Louie S, Cross CE, Motchnik P, Halliwell B (1993). Antioxidant protection against hypochlorous acid in human plasma. J Lab Clin Med.

[318] Colombo G, Clerici M, Giustarini D, Portinaro N, Badalamenti S, Rossi R, Milzani A, Dalle-Donne I (2015). A central role for intermolecular dityrosine cross-linking of fibrinogen in high molecular weight advanced oxidation protein product (AOPP) formation. Biochim Biophys Acta.

[319] Barrett AJ, Starkey PM (1973). The interaction of α2-macroglobulin with proteinases. Characteristics and specificity of the reaction, and a hypothesis concerning its molecular mechanism. Biochem J.

[320] Carrell RW, Jeppsson JO, Laurell CB, Brennan SO, Owen MC, Vaughan L, Boswell DR (1982). Structure and variation of human alpha 1-antitrypsin. Nature.

[321] He J, Turino GM, Lin YY (2010). Characterization of peptide fragments from lung elastin degradation in chronic obstructive pulmonary disease. Exp Lung Res.

[322] Segelmark M, Persson B, Hellmark T, Wieslander J (1997). Binding and inhibition of myeloperoxidase (MPO): a major function of ceruloplasmin?. Clin Exp Immunol.

[323] Chapman ALP, Mocatta TJ, Shiva S, Seidel A, Chen B, Khalilova I, Paumann-Page ME, Jameson GNL, Winterbourn CC, Kettle AJ (2013). Ceruloplasmin Is an Endogenous Inhibitor of Myeloperoxidase. J Biol Chem.

[324] Sokolov AV, Kostevich VA, Gorbunov NV, Grigorieva DV, Gorudko IV, Vasilyev VB, Panasenko OM (2018). A link between active myeloperoxidase and chlorinated ceruloplasmin in blood plasma of patients with cardiovascular diseases. Med Immunol.

[325] Vlasova II, Sokolov AV, Kostevich VA, Mikhalchik EV, Vasilyev VB (2019). Myeloperoxidase-induced oxidation of albumin and ceruloplasmin: role of tyrosines. Biochem.

[326] Hazell LJ, Stocker R (1993). Oxidation of low-density lipoprotein with hypochlorite causes transformation of the lipoprotein into a high-uptake form for macrophages. Biochem J.

[327] Hazell LJ, Arnold L, Flowers D, Waeg G, Malle E, Stocker R (1996). Presence of hypochlorite-modified proteins in human atherosclerotic lesions. J Clin Invest.

[328] Witztum JL, Steinberg D (1991). Role of oxidized low density lipoprotein in atherogenesis. J Clin Invest.

[329] McCall MR, Carr AC, Forte TM, Frei B (2001). Ldl modified by hypochlorous acid is a potent inhibitor of lecithin-cholesterol acyltransferase activity. Arterioscler Thromb Vasc Biol.

[330] Clark RA, Szot S, Williams MA, Kagan HM (1986). Oxidation of lysine side-chains of elastin by the myeloperoxidase system and by stimulated human neutrophils. Biochem Biophys Res Commun.

[331] Beck-Speier I, Leuschel L, Luippold G, Maier KL (1988). Proteins released from stimulated neutrophils contain very high levels of oxidized methionine. FEBS Lett.

[332] Domigan NM, Charlton TS, Duncan MW, Winterbourn CC, Kettle AJ (1995). Chlorination of Tyrosyl residues in peptides by myeloperoxidase and human neutrophils. J Biol Chem.

[333] Vossmann M, Kirst M, Ludolfs D, Schreiber M (2008). West Nile virus is neutralized by HOCl-modified human serum albumin that binds to domain III of the viral envelope protein E. Virology.

[334] Schoonbroodt S, Legrand-Poels S, Best-Belpomme M, Piette J (1997). Activation of the NF-κB transcription factor in a T-lymphocytic cell line by hypochlorous acid. Biochem J.

[335] Xie K, Bunse C, Marcus K, Leichert LI (2019). Quantifying changes in the bacterial thiol redox proteome during host-pathogen interaction. Redox Biol.

[336] Sies H (2017). Hydrogen peroxide as a central redox signaling molecule in physiological oxidative stress: oxidative eustress. Redox Biol.

[337] Marinho HS, Real C, Cyrne L, Soares H, Antunes F (2014). Hydrogen peroxide sensing, signaling and regulation of transcription factors. Redox Biol.

[338] Bauer G (2018). HOCl and the control of oncogenesis. J Inorg Biochem.

[339] Bechtel W, Bauer G (2009). Catalase protects tumor cells from apoptosis induction by intercellular ROS signaling. Anticancer Res.

[340] Zhou J, Wang Q, Ding Y, Zou M-H (2015). Hypochlorous acid via peroxynitrite activates protein kinase Cθ and insulin resistance in adipocytes. J Mol Endocrinol.

[341] Mütze S, Hebling U, Stremmel W, Wang J, Arnhold J, Pantopoulos K, Mueller S (2003). Myeloperoxidase-derived hypochlorous acid antagonizes the oxidative stress-mediated activation of iron regulatory protein 1. J Biol Chem.

[342] Midwinter RG, Vissers MC, Winterbourn CC (2001). Hypochlorous acid stimulation of the mitogen-activated protein kinase pathway enhances cell survival. Arch Biochem Biophys.

[343] Vile GF, Rothwell LA, Kettle AJ (1998). Hypochlorous acid activates the tumor suppressor protein p53 in cultured human skin fibroblasts. Arch Biochem Biophys.

[344] Fu X, Kao JLF, Bergt C, Kassim SY, Huq NP, Avignon A, Parks WC, Mecham RP, Heinecke JW (2004). Oxidative cross-linking of tryptophan to glycine restrains matrix metalloproteinase activity: specific structural motifs control protein oxidation. J Biol Chem.

[345] Eiserich JP, Baldus S, Brennan M-L, Ma W, Zhang C, Tousson A, Castro L, Lusis AJ, Nauseef WM, White CR, Freeman BA (2002). Myeloperoxidase, a leukocyte-derived vascular NO oxidase. Science.

[346] Palmer LJ, Cooper PR, Ling MR, Wright HJ, Huissoon A, Chapple ILC (2012). Hypochlorous acid regulates neutrophil extracellular trap release in humans. Clin Exp Immunol.

[347] Björnsdottir H, Welin A, Michaëlsson E, Osla V, Berg S, Christenson K, Sundqvist M, Dahlgren C, Karlsson A, Bylund J (2015). Neutrophil NET formation is regulated from the inside by myeloperoxidase-processed reactive oxygen species. Free Radic Biol Med.

[348] Metzler KD, Goosmann C, Lubojemska A, Zychlinsky A, Papayannopoulos V (2014). A myeloperoxidase-containing complex regulates neutrophil elastase release and actin dynamics during NETosis. Cell Rep.

[349] Karimi M, Ignasiak MT, Chan B, Croft AK, Radom L, Schiesser CH, Pattison DI, Davies MJ (2016). Reactivity of disulfide bonds is markedly affected by structure and environment: implications for protein modification and stability. Sci Rep.

